# Activation of Clustered IFNγ Target Genes Drives Cohesin-Controlled Transcriptional Memory

**DOI:** 10.1016/j.molcel.2020.10.005

**Published:** 2020-11-05

**Authors:** Wojciech Siwek, Sahar S.H. Tehrani, João F. Mata, Lars E.T. Jansen

**Affiliations:** 1 Department of Biochemistry, University of Oxford, Oxford OX1 3QU, UK; 2 Instituto Gulbenkian de Ciência, 2780-156 Oeiras, Portugal

**Keywords:** epigenetics, transcriptional memory, immunological priming, interferon γ, *GBP5*, stochastic gene expression, cohesin, gene regulation, transcription, signaling

## Abstract

Cytokine activation of cells induces gene networks involved in inflammation and immunity. Transient gene activation can have a lasting effect even in the absence of ongoing transcription, known as long-term transcriptional memory. Here we explore the nature of the establishment and maintenance of interferon γ (IFNγ)-induced priming of human cells. We find that, although ongoing transcription and local chromatin signatures are short-lived, the IFNγ-primed state stably propagates through at least 14 cell division cycles. Single-cell analysis reveals that memory is manifested by an increased probability of primed cells to engage in target gene expression, correlating with the strength of initial gene activation. Further, we find that strongly memorized genes tend to reside in genomic clusters and that long-term memory of these genes is locally restricted by cohesin. We define the duration, stochastic nature, and molecular mechanisms of IFNγ-induced transcriptional memory, relevant to understanding enhanced innate immune signaling.

## Highlights

• IFNγ induces transcriptional memory for up to 14 days in cycling cells
• Transcription during priming increases probability to reinduce
• Changes in local chromatin structure at primed genes are maintained short term
• IFNγ-induced memory in gene clusters is locally controlled by cohesin

## Graphical Abstract

**Figure:**
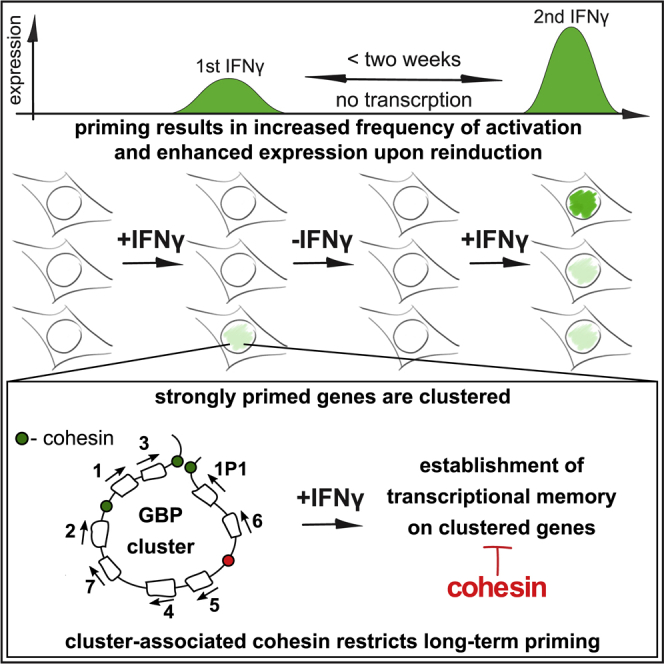

## Teaser

Siwek et al. find that IFNγ-induced transcriptional memory stably propagates through up to 14 cell division cycles. It manifests through an increased probability of primed cells to engage in target gene expression. The strongest memorized genes reside in two genomic clusters for which establishment of transcriptional memory is selectively restricted by cohesin.

## Introduction

Maintenance of gene expression states is essential for development and health. Local chromatin structure is implicated in preserving the transcriptional status of genes through cell division and cell lineages (Steffen and Ringrose, 2014). A role of *cis*-acting chromatin is most clearly defined in the maintenance of silent genes. During silencing, chromatin-associated proteins, modifications of DNA, and histones engage in self-propagating chromatin feedback loops that promote DNA compaction and restrict access for transcription factors (Gómez-Rodríguez and Jansen, 2013; Margueron and Reinberg, 2010). Active transcription can be maintained long term as well. However, in this case, self-propagation typically occurs through the self-amplified maintenance of *trans*-acting transcription factors that keep target genes expressed; for instance, during cellular differentiation (Grosschedl, 2013; Hoyler et al., 2012; Williams and Rudensky, 2007). Such cytoplasmic inheritance of transcription factors is arguably the most dominant form of epigenetic memory (Ptashne, 2011).

Whether *cis*-acting factors (e.g., local chromatin structure) contribute to maintenance of active transcriptional states remains unclear. Uncoupling the mechanisms that contribute to maintenance of transcription from those that contribute to transcription itself is challenging. However, there are instances of transcriptional memory where priming of gene activation can be maintained in the absence of the initial signal and, importantly, in the absence of ongoing transcription. Examples include sugar metabolism in *Saccharomyces cerevisiae* (Acar et al., 2005; Zacharioudakis et al., 2007), ecdysone response in *Drosophila melanogaster* (Pascual-Garcia et al., 2017), heat response in *Arabidopsis thaliana* (Lämke et al., 2016), and nuclear transfer in *Xenopus laevis* (Ng and Gurdon, 2005). In all of these cases, a primed state of transcription is maintained after the initial signal subsides.

An emerging paradigm for long-term transcriptional memory in mammalian cells is the primed response to cytokines (D’Urso and Brickner, 2017), which results in transient but reversible expression of pro-inflammatory and innate immune genes (Kamada et al., 2018; Light et al., 2013). When primed, cells maintain a memory of interferon exposure even in the apparent absence of target gene expression. This poised state is revealed upon a second interferon pulse, resulting in enhanced expression of a subset of genes (Gialitakis et al., 2010; Light et al., 2013). Therefore, interferon signaling offers an opportunity to dissect the mechanisms underlying memory of transcription and identify local chromatin-based contributors to memory.

Moreover, interferon-induced transcriptional memory in mammals may relate to the broader physiological phenomenon of trained immunity. This is an adaptive form of innate immunity where an organism, when exposed to a pathogen and triggering an innate immune response, retains a poised physiological state for weeks or months, resulting in an enhanced reaction upon a second exposure to the same or even entirely distinct insult (Netea et al., 2020). Striking examples of this phenomenon include enhanced resistance to *Staphylococcus aureus* after fungus-derived glucan treatment (Di Luzio and Williams, 1978; Marakalala et al., 2013) or hyperactivated anti-microbial effector genes after priming of macrophages with lipopolysaccharide (LPS) (Foster et al., 2007).

Interferon-mediated transcriptional memory has direct implications for enhanced innate immunity at the cell-autonomous level (e.g., resulting in an enhanced response to intracellular pathogens; Kamada et al., 2018; Sturge and Yarovinsky, 2014) and at the organismal level (Yao et al., 2018). Maintenance of a poised state to interferon may be one of the underlying mechanisms explaining trained immunity, and understanding the molecular nature of long-term transcriptional memory is therefore critical to advance our understanding of memory of innate immunity. However, studying transcriptional memory in the context of immunity poses challenges. For instance, priming of macrophages, key players in innate immunity, results not only in transient gene activation but also in sustained rewiring of transcriptional programs, enhancer activity, and lineage-specific transcription factor activation (Kang et al., 2017; Ostuni et al., 2013; Qiao et al., 2016). Therefore, in a physiological context, it is difficult to distinguish transient poised states from cellular differentiation. Interferon γ (IFNγ)-induced transcriptional memory has been established previously in HeLa cells. By using a non-hematopoietic cell type, we can avoid the confounding effects of lineage-specific transcription factor activation and therefore uncouple IFN-induced gene expression and memory from cellular differentiation.

Early work showed that a specific target gene, *HLA-DRA*, displays enhanced IFNγ-induced expression in cells that were primed previously by the same cytokine. Histone H3K4 dimethylation and retention of RNA polymerase II (RNA PolII) on promoters have been identified as molecular signatures that are retained in chromatin for up to 2 days after priming (Gialitakis et al., 2010; Light et al., 2013). Additionally, Nup98, a component of the nuclear pore (Light et al., 2013), as wells the CDK8+ mediator complex (D’Urso et al., 2016) are implicated in IFNγ transcriptional memory. More recently, IFNβ and IFNγ priming in mouse fibroblast and macrophages, respectively, has been shown to cause a similar memory effect. In this case, retention of the H3.3 replacement histone variant and histone H3K36 trimethylation were reported to be maintained on memory genes after removal of the cytokines (Kamada et al., 2018). However, in this context, retention of promoter-bound RNA polymerase was not detected.

Despite identification of several chromatin-associated factors required for transcriptional memory, there are many unanswered questions. Here we define the nature of IFNγ-primed genes and the duration of memory. We address the population dynamics of memory and the role of active transcription and local chromatin structure in maintenance of the primed state.

## Results and Discussion

### IFNγ Primes *GBP5* for Long-Term Transcriptional Memory

**Figure 1:**
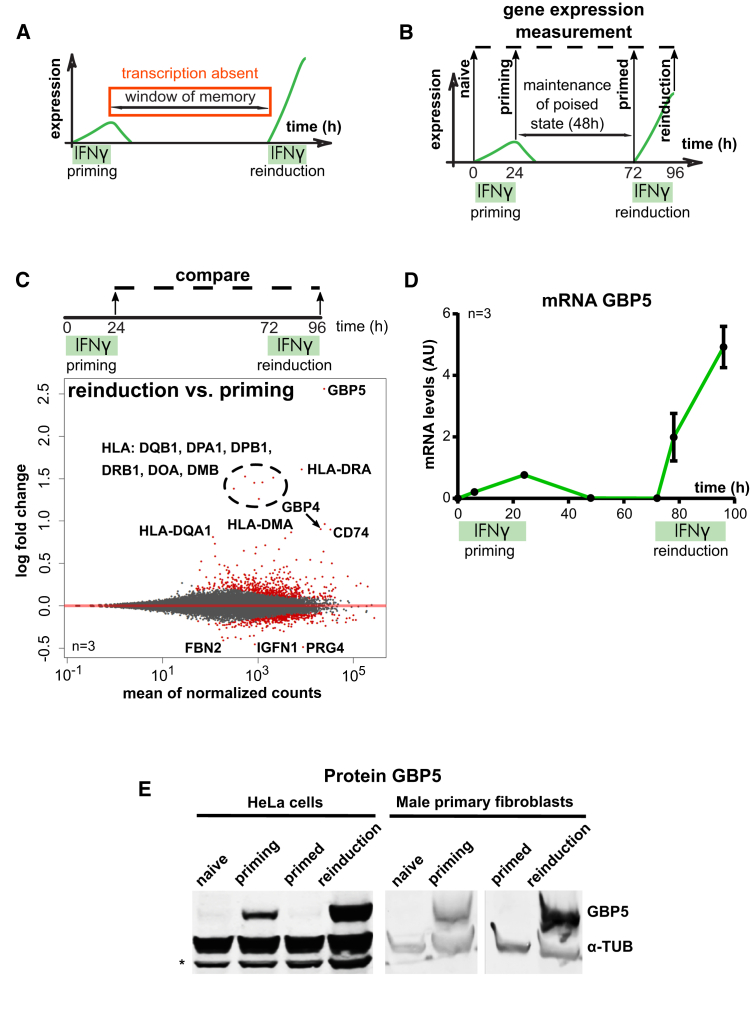
Long-Term Transcriptional Memory of Genes Following Priming with IFNγ (A) Principle of cytokine-induced transcriptional memory. (B) Experimental outline of the transcriptional memory experiment. (C) Plot showing differential rates of reinduction relative to priming as measured by RNA-seq. Average read counts for three replicate experiments, as outlined in (B), were assembled for each gene. The log2 fold change of the gene read count following reinduction over those following priming is plotted. “0” indicates no change, whereas positive values indicate increased expression upon reinduction. Data were ranked according to the mean expression level for both of the conditions and all replicates. Genes that show strong transcriptional memory or tolerance (reduced expression upon reinduction) are labeled. Red dots represent genes with a p-adj value below 0.1. (D) HeLa cells were primed and reinduced according to the regimen outlined in (B). *GBP5* mRNA levels were quantified by RT-qPCR and normalized to *ACTB* expression. Error bars, SD; n = 3 biological replicates. (E) HeLa cells and human male primary fibroblasts were subjected to the IFNγ treatment regimen outlined in (B), processed for western blotting, and probed for GBP5 protein expression. α-Tubulin (α-TUB), loading control; ^∗^, cross-reacting band. See also Figure S1.

We aimed to identify all human genes that show long-term transcriptional memory following IFNγ priming in HeLa cells. Memory is defined as enhanced expression of target genes upon a second exposure to IFNγ (conceptualized in Figure 1A). We primed HeLa cells with IFNγ and allowed cells to recover for 48 h before reinduction to IFNγ (Figure 1B). In agreement with previous reports (Gialitakis et al., 2010; Light et al., 2013), we found *HLA-DRA* to be expressed 5-fold higher in IFNγ-primed relative to naive cells upon IFNγ re-exposure (Figure S1A). We used this IFNγ priming and reinduction regimen to unbiasedly discover all genes that behave in a manner analogous to *HLA-DRA*. We treated HeLa cells with IFNγ as in Figure 1B and analyzed transcript levels in naive cells, cells undergoing priming, primed, and reinduced cells by RNA sequencing. As expected, when comparing priming with naive cells, we observed numerous IFNγ-induced gene expression changes: activation (3,531 genes above 0.5 log2 fold change cutoff, adjusted p value [p-adj] < 0.05) and inhibition (2,117 genes below −0.5 log2 fold change cutoff, p-adj < 0.05) (STAR Methods; Figure S1B; Table S1). Importantly, we identified a small subset of IFNγ-induced genes that show transcriptional memory (as defined in Figure 1A) when comparing reinduced with priming transcriptomes (28 genes above 0.5 log2 fold change cutoff, p-adj < 0.05) (Figure 1C; Table S2), indicating that memory is not simply a consequence of signaling.

Among these are the previously established *HLA-DRA* gene (Figure S1A; Gialitakis et al., 2010; Light et al., 2013) and other major histocompatibility complex (MHC) class II genes. We found the gene encoding guanylate binding protein 5 (GBP5), an activator of inflammasome assembly (Shenoy et al., 2012), to display the strongest reinduction following initial priming (Figure 1C). We validated this observation by RT-qPCR measurements of *GBP5* mRNA and determined an ∼10-fold increase in expression of this target during reinduction relative to priming (Figure 1D). Enhanced *GBP5* expression upon stimulation of primed cells was also detected at the protein level by western blot (Figure 1E). Importantly, long-term transcriptional memory is not restricted to cancer cells because we observed the same phenomenon in primary male fibroblasts (Figure 1E). However, priming is not universal among cultured human cells because HCT116 colon cancer cells and non-transformed retinal pigment epithelium (RPE-1) cells did not show transcriptional memory of *GBP5* despite responding to IFNγ (Figure S1C). This suggest that, although IFNγ signaling and memory are broad phenomena, they are not hard wired, at least for *GBP5*, indicating that components involved in propagation of transcriptional memory are subject to regulation.

### Memory of Prior IFNγ Induction Is Reversible but Persists for up to 14 Days in Continuously Cycling Cells

**Figure 2:**
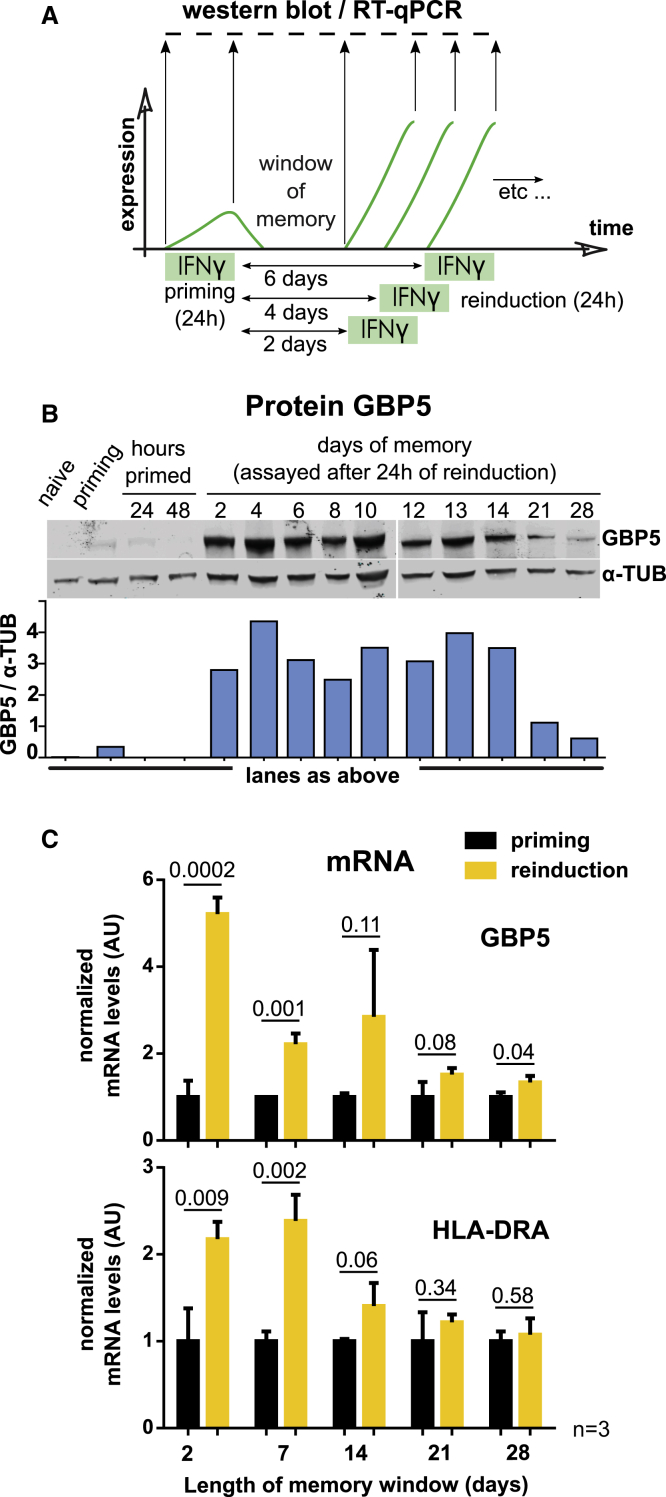
Memory of Prior IFNγ Induction Is Reversible but Persists for up to 14 Days in Continuously Cycling Cells (A) Scheme outlining the experiment to determine the duration of IFNγ-mediated transcriptional memory. (B) Cells subjected to the IFNγ treatment regimen outlined in (A) were processed for western blotting and probed for GBP5 protein expression. α-TUB, loading control. (C) Cells as in (B) but processed for RT-qPCR of *GBP5* and *HLA-DRA* mRNA. Expression levels were normalized to *ACTB* and internally to cells after priming. Error bars, SD; n = 3 biological replicates; numbers represent the p values.

One of the key features of transcriptional memory is its reversibility because this distinguishes it from a permanently altered transcriptional profile, such as cellular differentiation. Previous efforts have shown that priming by IFNγ lasts for at least 48 h (Gialitakis et al., 2010; Light et al., 2013) or even up to 8 days following priming by IFNβ in mouse cells (Kamada et al., 2018). However, the full extent of memory and the timing of its eventual loss have not been determined. To this end, we primed HeLa cells with IFNγ for 24 h and left cells to proliferate for an increasing number of days before reinduction (Figure 2A). Enhanced GBP5 protein expression upon reinduction was evident for up to 14 days after priming but was lost at later time points (Figure 2B). During this time, cells are doubling daily and are passaged continuously, equaling up to 2^14^ cells. Similarly, *GBP5* and *HLA-DRA* mRNA levels followed this trend; we detected enhanced activation for at least 7 days after priming (Figure 2C). This indicates that memory persists through many mitotic cell division cycles, at least for the *GBP5* and *HLA-DRA* genes. Such mitotic stability of the primed state is unlikely to be a consequence of passive dilution of a factor induced during priming. Instead, an active positive feedback mechanism may be at play to maintain memory over such timescales. Importantly, the primed state is ultimately lost by 3 weeks of culture, indicating that priming is not a differentiation state involving rewiring of the transcriptional network.

### Priming Results in Increased Frequency of *GBP5* Activation and Enhanced Expression upon Reinduction

**Figure 3:**
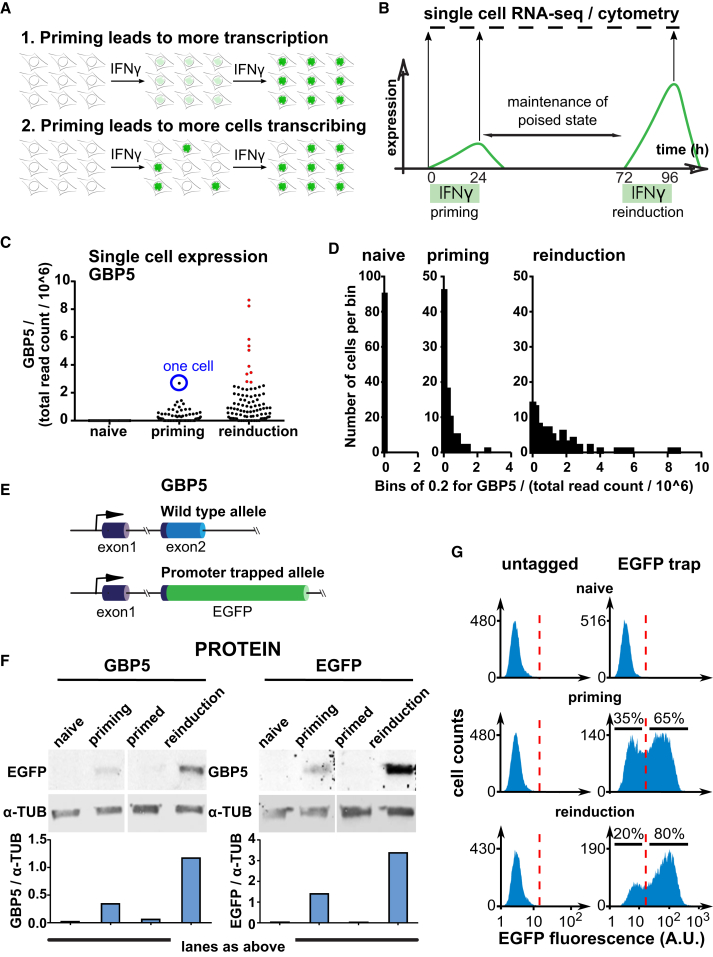
Priming Results in Increased Frequency of Activation and Enhanced *GBP5* Expression upon Reinduction (A) Alternative solutions for achieving IFNγ transcriptional memory. (B) Scheme describing a single-cell RNA-seq transcriptional memory experiment to distinguish between the alternatives from (A). (C) Representation of the single-cell RNA-seq data from HeLa cells for the *GBP5* gene from the experiment shown in (B). Each dot represents the expression level of the *GBP5* gene for one cell in the naive (N = 90), priming (N = 89), and reinduction state (N = 91). (D) Binned representation of the single-cell RNA-seq data for the *GBP5* gene from the experiment shown in (C). (E) Scheme outlining the *GBP5* promoter trap cell line, in which *EGFP* was inserted into exon 2 downstream of the *GBP5* translation start site. One allele was targeted, and the remaining allele remained unperturbed. Purple, 5′ UTR; blue, *GBP5* coding sequence. (F) EGFP::GBP5 cells were subjected to the IFNγ treatment regimen outlined in Figure 1B, processed for fluorescence western blotting, and probed for GBP5 and EGFP expression. α-TUB, loading control. Tubulin-normalized fluorescence intensities are plotted. (G) EGFP::GBP5 cells were subjected to the IFNγ treatment regimen as outlined in (B) and processed for cytometry. Cell frequencies as a function of EGFP fluorescence intensity are plotted. Red dotted lines are fiducial marks based on untagged cells and are used to define the cutoff for cell percentages expressing or not expressing. See also Figure S2.

Next we aimed to characterize the cellular basis of transcriptional memory. We explored two possible explanations for enhanced transcriptional output following IFNγ reinduction. Priming can lead to increased transcription of the *GBP5* gene in the next round of stimulation (Figure 3A, hypothesis 1) and/or more cells participating in transcription upon reinduction (hypothesis 2). We found no effect of IFNγ on selective cell proliferation or lethality, excluding a trivial explanation of cell selection (Figures S1D and S1E). To distinguish between the possibilities in Figure 3A, we performed single-cell RNA sequencing (RNA-seq) during the course of IFNγ induction and reinduction (Figure 3B). Consistent with the bulk RNA-seq data, single-cell mRNA levels showed that, on average, IFNγ priming resulted in enhanced expression of the *GBP5* and *HLA-DRA* genes upon second exposure, whereas a constitutive control gene, *ACTB*, remained unaltered throughout the experiment (Figures 3C, S1F, and S1G). However, induction and reinduction were not uniform across the population. The per-cell transcriptional output of *GBP5* was increased upon re-exposure to IFNγ, but only in 10 of 91 cells was expression boosted beyond the level observed during priming (Figure 3C, labeled with red dots); in many cells, *GBP5* was not detectably induced. To further illustrate this, we replotted the data in Figure 3C by binning cells based on their *GBP5* expression level. In ∼52% of cells, *GBP5* was not induced during priming, whereas non-responsive cells represented only a minor (∼15%) fraction following reinduction (Figure 3D). These data also indicate that stronger re-expression of *GBP5* did occur to some extent. Approximately 35% of total transcriptional output comes from cells expressing higher levels during reinduction than during priming (red cells in Figure 3C), consistent with hypothesis 1. However, an increase in the population of cells that express *GBP5* (hypothesis 2) appears to be responsible for the majority of the enhanced overall *GBP5* output upon reinduction, constituting long-term transcriptional memory.

To explore this phenomenon further, we inserted an EGFP-based promoter trap at one of the genomic HeLa *GBP5* alleles that allowed us to monitor GBP5 output on a per-cell basis (Figure 3E). We confirmed by flow cytometry that EGFP::GBP5 expression is specifically induced by IFNγ but not IFNα or IFNβ, as reported previously for *GBP5* (Figure S1H; Degrandi et al., 2007; Krapp et al., 2016). Priming of EGFP::GBP5 cells resulted in enhanced reinduction of *EGFP* by IFNγ, confirming preservation of transcriptional memory for the EGFP-tagged allele and the wild-type *GBP5* allele in the same cells, as measured by western blot (Figure 3F). Cytometry-based analysis of cells showed that, during priming, 65% of cells detectably responded to IFNγ, whereas this number increased substantially to 80% upon reinduction (Figure 3G). Notably, the per-cell EGFP fluorescence did not increase significantly. These results are consistent with the single-cell RNA-seq data and indicate that, although transcriptional output per cell may be enhanced to some extent, the dominant mechanism of IFNγ priming of *GBP5* is to render transcription upon second expose more likely.

### Establishment of *GBP5* Transcriptional Memory Correlates with Local Transcriptional Output

The population dynamics of *GBP5* expression, as revealed by cytometry in Figure 3G, indicated that not all cells express, even upon repeated activation. We consider two possibilities. (1) cells have an inherent variability in their *GBP5* response to IFNγ. For instance, this may be the case when there are clonal genetic differences in the cell population. (2) All cells have an equal but limited probability to induce *GBP5*, and exposure to IFNγ shifts this probability toward an increased chance of expressing upon later exposure. To explore these possibilities, we tracked cell populations that responded to priming or not and monitored their behavior during a second stimulation. Single-cell RNA-seq data of IFNγ-treated cells (Figure 3B) showed that all cells in the population (even when not activating EGFP::GBP5) were uniformly responsive to IFNγ (Figure S2A), indicating that the variability in *GBP5* activation was not due to lack of IFNγ-mediated Janus kinase / Signal Transducer and Activator of Transcription (JAK/STAT) signaling in these cells. Following initial priming, roughly half of the cell population induced *GBP5* expression, as measured by EGFP reporter expression (Figure S2B). We then sorted all cells or the top 20% and bottom 20% of EGFP-expressing cells and allowed these to proliferate before restimulation. We found that cells that did not respond to priming still had the ability to express EGFP but at a lower probability than cells that expressed EGFP during priming. We conclude that, despite all cells responding to IFNγ (Figure S2A), whether cells express EGFP::GBP5 correlates with prior *GBP5* activation. Importantly, this relationship is not deterministic but, rather, manifests as a change in probabilities. This means that, although priming correlates with reinduction, the reverse also happens, but at lower probability; i.e., cells that express during priming can become silent during reinduction (Figure S2B). Combined, these results suggest that memory of *GBP5*, as manifested by increased frequency of expression, is a consequence of expression during priming. This is akin to promoter-enhancer interactions, where enhancers have been shown to increase the probability and robustness but not the extent of gene expression (Fiering et al., 2000; Pascual-Garcia et al., 2017; Perry et al., 2010). Furthermore, resent studies have shown transcription to occur in episodic bursts. Enhancer activation of genes results not in increased transcription amplitude or longer bursts but, rather, in an increased frequency of bursts (Fukaya et al., 2016). Similarly, a recent study of transcriptional memory in yeast showed priming to result primarily in an increased frequency in transcription engagement, not an increased rate of transcription (Bheda et al., 2020). We suspect that the population-level priming we observed for *GBP5* may be related and represents a general phenomenon in transcriptional memory.

### Ongoing Transcription Is Not Required for Maintenance of IFNγ Transcriptional Memory

**Figure 4:**
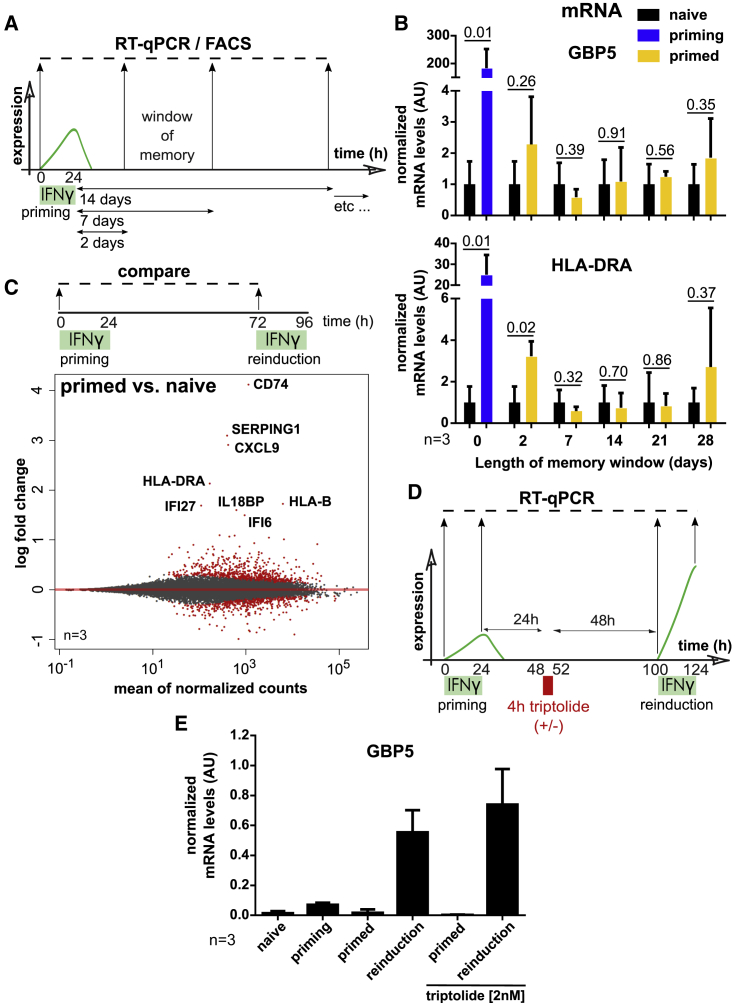
*GBP5* and *HLA-DRA* mRNA Levels in Primed Cells Return to the Pre-induced Levels of Naive Cells, and Ongoing Transcription Is Not Required for Maintenance of Transcriptional Memory (A) Scheme to measure long-term transcriptional output of memory genes following priming. (B) HeLa cells were subjected to the IFNγ treatment regimen outlined in (A) and processed for RT-qPCR of *GBP5* and *HLA-DRA* mRNA. Signals were normalized to *ACTB* expression and internally to naive cells. Error bars, SD; n = 3 biological replicates; numbers represent the p values. (C) Representation of processed RNA-seq data in HeLa cells analogous to data in Figure 1C but for primed over naive cells. (D) Outline of the triptolide-based RNA Pol II inhibition experiment. (E) HeLa cells were subjected to the IFNγ and triptolide treatment regimen outlined in (D) and processed for RT-qPCR of *GBP5* mRNA. Signals were normalized to *ACTB* expression. Error bars, SD; n = 3 biological replicates. See also Figure S3.

One possible mechanism for the memory of transcription through successive cell division cycles is continued low-level expression of the target genes, which is contrary to the preconception that transcription fully shuts down after IFNγ removal. To address this, we measured long-term transcriptional output of memory genes 2, 7, 14, 21, and 28 days following priming (Figure 4A). Although overall transcription levels are exceedingly low during the window of memory compared with the priming stage, we could still detect 2- and 3-fold higher levels of *GBP5* and *HLA-DRA* mRNA, respectively, after 2 days compared with the low basal level of naive cells (Figure 4B). However, by day 7, we could not detect any difference, and transcript levels fully returned to baseline despite continued memory of *GBP5* and *HLA-DRA* priming (Figure 2). We confirmed these observations in the EGFP::GBP5 reporter line, where we observed low but detectable expression of EGFP above the naive baseline 2 days but not 7 days following cytokine removal (Figure S3A).

Although we do not find evidence of continued target gene expression during memory, it is possible that the primed state of memory genes is maintained by continued expression of secondary genes that may drive reinduction upon IFNγ exposure. We re-analyzed the RNA-seq dataset as in Figure 1C but now plotted the differential expression of genes in primed versus naive cells (Figure 4C). In this scenario, most IFNγ-induced genes had returned to baseline. However, we did find a small subset of genes whose expression was still detectable 48 h after priming (93 genes above 0.5 log2 fold change cutoff, p-adj < 0.05) (Table S3), although the levels were dramatically lower than immediately following IFNγ induction (Figure S3B). Among these are *HLA-DRA*, as evident in Figure 4B (2 days after IFNγ); *CD74*, a chaperone stabilizing the peptide-free MHC class II complexes (Hiltbold and Roche, 2002); *SERPING1*, an inhibitor of the C1 complement pathway (Davis, 2004); and *CXCL9*, a chemokine involved in the inflammatory response (Egesten et al., 2007). However, none of these have an ascribed role in IFNγ signaling or target gene expression, making a continued transcription feedback loop driving memory unlikely.

To further test whether continued transcription is required to maintain memory, we inhibited transcription during the memory window, after priming but before reinduction (Figure 4D), using a brief treatment with triptolide, a fast-acting RNA polymerase II inhibitor (Vispé et al., 2009). We titrated the minimal amount needed to effectively block transcription to avoid non-specific effects (Figures S3C and S3D) and treated cells transiently for 4 h during the primed state (Figure 4D). RT-qPCR analysis of *GBP5* expression throughout the experiment showed that disruption of transcription had no bearing on *GBP5* reinduction (Figure 4E). We conclude that continuous global transcriptional output is not required for maintenance of transcriptional memory of *GBP5*. These observations indicate that other mechanisms are likely responsible for maintenance of the primed state.

### Short-Term Maintenance of H3.3, H3K4me2, and H3K79me2 on GBP Genes Following IFNγ Priming

**Figure 5:**
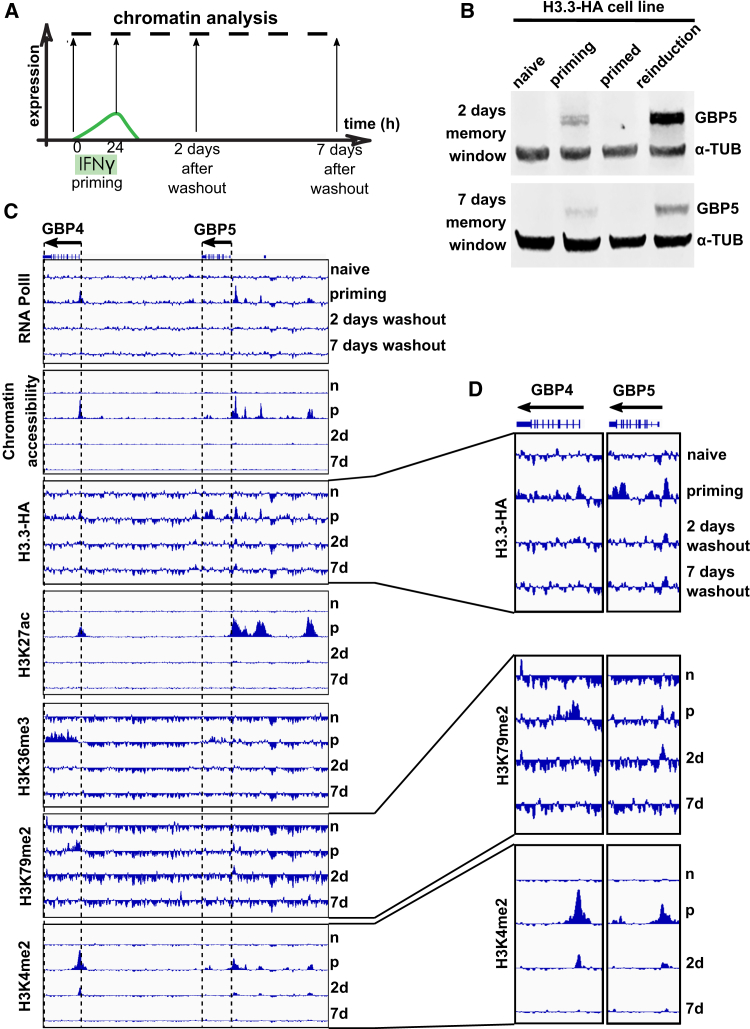
Short-Term Maintenance of H3.3, H3K4me2, and H3K79me2 on the *GBP5* and *GBP4* Genes following IFNγ Priming (A) Outline of the experiment to measure chromatin status following IFNγ-mediated priming. (B) HeLa cells ectopically expressing H3.3-SNAP-HA were subjected to the IFNγ treatment regimen outlined in Figure 1B with a 2- and 7-day window of memory, processed for western blotting, and probed for GBP5 protein expression. α-TUB, loading control. (C) Representation of data for processed chromatin accessibility (ATAC-seq) and occupancy of the indicated chromatin-associated factors (ChIPmentation) for the time points indicated in (A). Sequenced reads were mapped to the human genome (hg38), and coverage data are displayed with internal scaling between naive, primed, 2- and 7-day IFNγ washout samples. Two proximally positioned memory genes, *GBP4* and *GBP5*, are shown. (D) Enlarged presentation of the *GBP4* and *GBP5* genes.

We next determined whether chromatin features contribute to *GBP5* transcriptional memory in the absence of ongoing transcription. We analyzed chromatin structure and protein occupancy in naive cells during priming and at different time points after IFNγ washout in primed cells to assess chromatin maintenance (Figure 5A). We employed a HeLa cell line expressing a SNAP-hemagglutinin (HA)-tagged version of the H3.3 variant to assess H3.3 levels (Bodor et al., 2013; Ray-Gallet et al., 2011). We confirmed transcriptional memory of the *GBP5* gene 2 and 7 days after priming (Figure 5B), although the degree of long-term priming appears to be less pronounced compared with unmodified HeLa cells (Figures 1E and 2B). Using chromatin immunoprecipitation coupled to sequencing (ChIP-seq), we determined the occupancy of H3.3-HA (using the HA epitope) as well as RNA Pol II, histone H3 acetylation of lysine 27 (H3K27ac), trimethylation of lysine 36 (H3K36me3), dimethylation of lysine 79 (H3K79me2), and dimethylation of lysine 4 (H3K4me2) genome-wide. In addition, we determined global chromatin accessibility using ATAC-seq (Chen et al., 2016). As expected from active chromatin features, RNA Pol II, H3.3, and the above histone modifications accumulated during priming at *GBP5* and the neighboring *GBP4* gene (Figure 5C), correlating with opening of chromatin at the *GBP5* and *GBP4* promoters and induction of gene expression (Figures 1C–1E). However, we did not detect any appreciable maintenance of RNA Pol II, accessible chromatin, H3K27ac, or H3K36me3 on these genes (Figure 5C). Similarly, lack of RNA Pol II maintenance has been reported recently for IFNγ priming in mouse macrophages (Kamada et al., 2018). In contrast, we found low levels of H3.3-HA, H3K79me2, and H3K4me2 chromatin marks to be maintained on promoters of GBP genes for up to 2 days after IFNγ washout. Specifically, 2 days after priming, we detected low levels of H3.3-HA on the promoter of *GBP5* but not *GBP4* and H3K79me2 on the promoter of *GBP5* but not *GBP4*, and H3K4me2 for both genes (Figures 5C and 5D). A role of H3.3 has been suggested previously for IFNγ transcriptional memory in mouse macrophages along with trimethylation of lysine 36 (Kamada et al., 2018), although we did not find significant retention of the latter mark in primed cells. H3K4me2 is of interest because it has been reported independently in several studies (Gialitakis et al., 2010; Light et al., 2013). Marking active promoters, it possibly plays a role in maintenance of a poised state, at least in the short term. In line with such a role, this modification has also been shown to be important in *Dictyostelium discoideum* gene expression memory (Muramoto et al., 2010) and mitotic inheritance of gene expression states in *Xenopus laevis* nuclear transfer experiments (Ng and Gurdon, 2008). Moreover, a mechanism of maintenance of this mark involving the SET3C methyltransferase complex has been demonstrated in heritable maintenance of a poised *INO1* gene in yeast (D’Urso et al., 2016). Nevertheless, the short maintenance of these chromatin features suggests that, although they may contribute to maintenance of the primed state, they are not the sole factors and unlikely to be key factors driving memory of IFNγ priming, at least for GBP genes.

### The Two Major Classes of Genes that Are Primed for Re-expression, GBPs and HLAs, Reside in Genomic Gene Clusters

Analysis of the top 28 genes that display transcriptional memory (Figure 1C) revealed that primed genes with the strongest reactivation phenotype in HeLa cells belong to two main gene families: GBPs and HLAs. These gene families form two separate gene clusters on chromosomes 1p22 and 6p21, respectively. The majority of IFNγ-induced genes in the HLA cluster and 3 of 6 IFNγ-induced genes in the GBP cluster show transcriptional memory (Figures S4A and S4B). Moreover, genes of the GBP cluster fall within a single topologically associating domain (TAD) (Figure S5A; Rao et al., 2014). However, not all IFNγ-primed genes appear in clusters. *CD74* on chromosome 5q33 is an example of an isolated gene showing IFNγ-mediated transcriptional memory (Figure S4C). Local chromatin domains often show regulatory mechanisms spanning several genes; for instance, at the classic β-globin gene cluster, where a proximal locus control region governs temporally and developmentally regulated expression of globin genes (Noordermeer and de Laat, 2008). Furthermore, another report showed that long non-coding RNAs control expression of genes in a chemokine cluster (Fanucchi et al., 2019). Therefore, despite the lack of long-term maintenance of changes in local chromatin structure (Figure 5), it is possible that longer-range chromatin organization has a selective effect on these clusters and regulates priming and memory.

### Cohesin Inhibits Establishment but Not Maintenance of IFNγ Memory for Most Genes in the GBP and HLA Clusters

To explore the role of long-range chromatin interactions, we manipulated the levels of cohesin, the principal organizer of local chromatin interactions (Hadjur et al., 2009; Kagey et al., 2010; Rao et al., 2017). We removed functional cohesin specifically during different stages of IFNγ priming and memory, using a HeLa cell line in which both alleles of the essential Kleisin subunit *SCC1/RAD21* of the cohesin ring are tagged with an auxin-inducible degron (AID) tag (Wutz et al., 2017; Figure S5B). Combined with the *Oryza sativa*-derived TIR1 E3 ligase, this degron results in rapid protein depletion in the presence of auxin (indole-3-acetic acid [IAA]; Nishimura et al., 2009). Based on SCC1-EGFP-AID fluorescence, we determined that auxin mediated depletion of SCC1 is complete in 3 h and fully recovers within 48 h upon IAA washout (Figure S5C).

**Figure 6:**
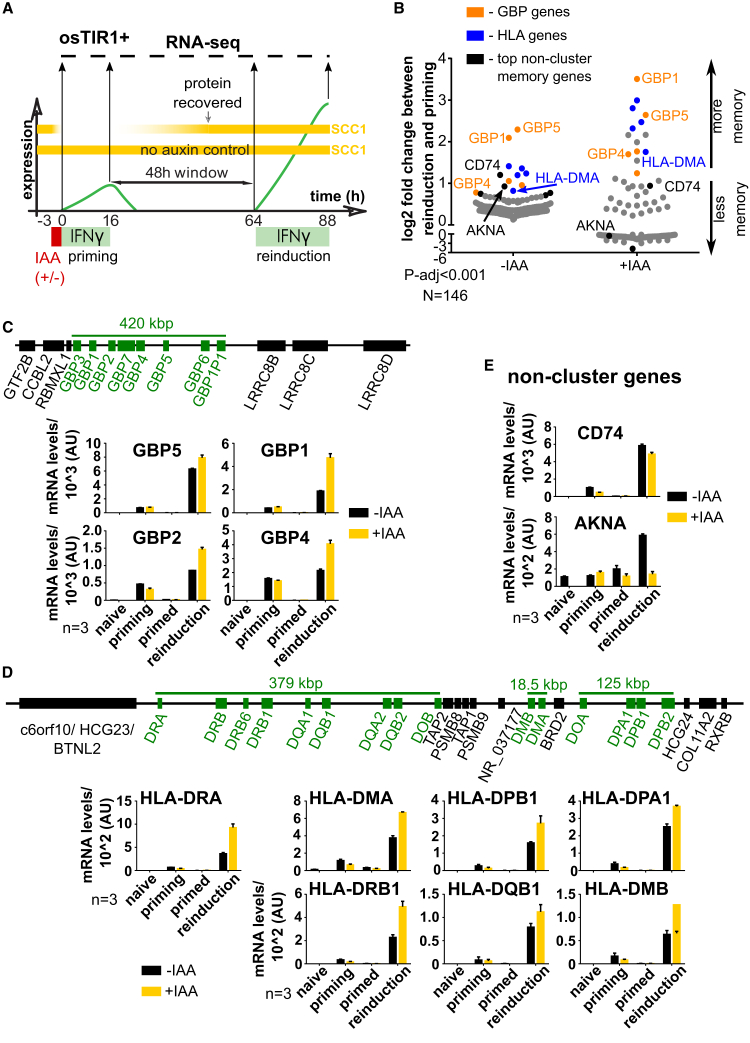
Cohesin Negatively Regulates Memory for Most Genes in the GBP and HLA Clusters but Not for Genes Outside of Those Clusters (A) Outline of a transcriptional memory experiment (analogous to Figure 1B) combined with auxin-mediated transient depletion of SCC1 during priming, analyzed by RNA-seq. (B) HeLa Kyoto SCC1-EGFP-AID osTIR1-positive cells were subjected to the IFNγ and auxin treatment regimen outlined in (A) and processed for RNA-seq. The Log2 fold change for memory genes between reinduction (with or without auxin) and priming (without auxin) was plotted. “0” indicates no memory. The genes shown were selected based on a p-adj value below 0.001 as determined by the DESeq2 software (Love et al., 2014). (C) Top: representation of the genomic structure of the GBP locus. Bottom: individual gene plots from the data described in (B). Error bars, SD; n = 3 biological replicates. (D) Top: representation of the genomic structure of the HLA locus. Bottom: data presented as in (C) but for the HLA cluster genes. (E) Data presented as in (C) but for the *CD74* and *AKNA* genes. See also Figures S4–S7.

To determine the role of cohesin in establishment of transcriptional memory, we depleted SCC1 in osTIR1-positive cells during IFNγ priming (Figure 6A) and collected samples for RNA-seq analogous to the transcriptional memory assay as in Figure 1B. Previous work reported that cohesin depletion has a minor effect on steady-state gene expression (Rao et al., 2017; Schwarzer et al., 2017; Wendt et al., 2008; Zuin et al., 2014). However, we found that, during IFNγ gene activation, cohesin loss has broader effects on transcription. 1,628 genes were affected positively and negatively (above 0.5 or below −0.5 log2 fold change cutoff, P-adj < 0.05) by acute short-term SCC1 depletion during IFNγ priming (Figure S5D). These effects are consistent with a recent report of cohesin depletion during LPS activation that showed many of the inducible genes to be affected (Cuartero et al., 2018). Despite these expected global effects, we found cohesin loss to uniquely affect IFNγ-primed gene clusters where the majority of the clustered memory genes (GBPs and HLAs) showed enhanced memory; i.e., stronger gene expression upon reinduction following IFNγ priming in the absence of SCC1 (Figure 6B). Although cohesin had no bearing on induction, reinduction was enhanced even though cohesin levels were restored to normal levels before the second exposure to IFNγ (Figures 6A, 6C, 6D, and S5C). In contrast, the majority of non-cluster-associated memory genes lost memory upon cohesin depletion or were unaffected (e.g., *CD74* and *AKNA*) (Figures 6B and 6E). The effect on transcriptional memory is specific to SCC1 depletion because IAA treatment of SCC1-EGFP-AID-tagged cells lacking the osTIR1 E3 ligase had no effect on any of the analyzed genes during any stage of IFNγ priming, memory, or reinduction (Figures S6A–S6D). Although all strongly expressed genes in the GBP and HLA clusters showed enhanced memory upon cohesin depletion, a minor subset of genes in the clusters that are expressed at much lower levels did not display such an effect of cohesin depletion (Figures S7A–S7D), indicating that cohesin control is most prominent for robustly expressed genes.

Among the top 15 primed genes we found 3 additional non-clustered genes: *GTPBP2*, *INHBE*, and *HIST1H2AC* (Figure S7E). We confirmed that, among these genes (along with *CD74* [Figure S4C] and *AKNA*), IFNγ-induced memory is restricted to the identified candidates with no effect on neighboring genes, with a few exceptions of more distal genes; i.e., *TMEM268* (proximal to *AKNA*) and *MARS* (proximal to *INHBE*) (Figure S7F). All of these non-cluster associated genes showed impaired memory establishment upon cohesin depletion, contrary to the cluster-associated GBP and HLA genes (Figures 6E, S6D, S7G, and S7H).

Next, to determine whether impaired GBP/HLA gene memory is specifically linked to cohesin loss during IFNγ priming, we primed cells in the presence of cohesin but removed cohesin during maintenance of the primed state (Figure S6E). Despite systemic loss of SCC1, removal of cohesin function during the memory window (as opposed to during priming) had no effect on maintenance of *GBP5* or *HLA-DRA* transcriptional memory (Figure S6F). These results indicate that SCC1 function is selectively required during the priming phase of long-term memory establishment but becomes dispensable when memory is established.

### Local, Intra-TAD Cohesin Binding Restricts Transcriptional Memory of the *GBP5* and *GBP1* Genes

**Figure 7:**
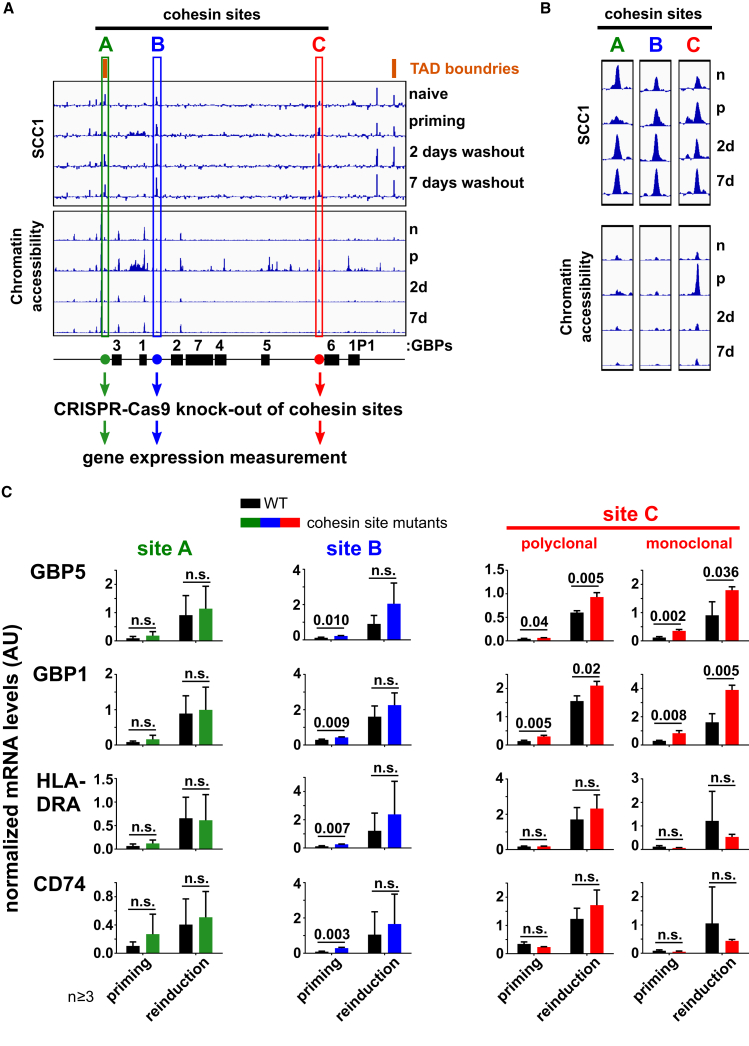
Cohesin Bound at the GBP Cluster Inhibits Establishment of Transcriptional Memory within the Cluster but Not for Distal Genes (A) Representation of processed data for occupancy of SCC1 (ChIPmentation) and chromatin accessibility (ATAC-seq; see also Figure 5C) during the IFNγ long-term memory assay described in Figure 5A. Data are plotted as in Figure 5C. The results are shown for the GBP cluster; TAD boundaries (Figure S5A) and prominent cohesin sites are indicated. (B) Enlarged presentation of three selected cohesin sites. (C) Approximately 1-kb regions encompassing cohesin-bound sites boxed as A, B, and C in (A) were deleted by CRISPR-Cas9. Two independent polyclonal populations were generated for site A, single polyclonal populations for sites B and C, and a monoclonal clone for site C. Mutant cells were subjected to the transcriptional memory experiment outlined in Figure 1B. mRNA levels were quantified by RT-qPCR for the indicated genes and normalized to *ACTB* expression. Error bars, SD; n = 3 (n = 6 for site A) biological replicates. n.s., non-significant (p > 0.05). See also Figure S7.

The selective effect of cohesin depletion during IFNγ priming on the maintenance of the primed stated led us to the hypothesis that cohesin controls local chromatin structure within the cluster, restricting long-term memory. However, loss of cohesin also has broad effects on gene expression (Figure S5D). It is therefore possible that the effect of cohesin depletion on clustered GBP and HLA genes is indirect. To distinguish between these possibilities, we mapped cohesin binding and locally manipulated its function. We focused on the GBP cluster as the principal locus of IFNγ-induced transcriptional memory. First we performed a ChIP-seq experiment for SCC1 during IFNγ stimulation and washout as outlined in Figure 5A. We identified several cohesin-bound sites, including at the boundaries of the previously identified TAD (Rao et al., 2014; Figures 7A and S5A). Among these, we found three prominent peaks within or immediately adjacent to the GBP genes that show IFNγ priming, which we designated sites: A, B, and C (Figures 7A and 7B). Although global cohesin positions were preserved throughout the experiment, and occupancy did not change considerably, particularly during memory (Figure S7I), we found that two of the sites in the GBP cluster were remodeled upon IFNγ stimulation. Site A on the TAD boundary showed loss of cohesin occupancy during priming, whereas site C within the cluster displayed enhanced chromatin accessibility (as measured by ATAC-seq) at this stage (Figures 7A and 7B). These findings indicate that cohesin is chromatin bound in the GBP cluster and that IFNγ priming results in local changes in cohesin binding or organization.

Next we determined whether local cohesin binding is functionally required for GBP gene priming. We performed CRISPR-Cas9 mutagenesis to selectively remove the three principal cohesin binding sites in the cluster. We created polyclonal CRISPR-targeted cell populations, deleting an ∼1-kb region encompassing each site, and determined the effect of the perturbation on transcriptional memory of the cluster-based *GBP1* and *GBP5* genes as well as *HLA-DRA* and a non-cluster memory gene, *CD74*. We found that removal of the TAD border (site A) or the adjacent intra-TAD (site B) cohesin sites has no significant effect on reinduction of any of the analyzed genes. However, removal of site C, which is also positioned within the TAD, resulted in enhanced priming and stronger reinduction of *GBP1* and *GBP5*. Site C coincides with a known CCCTC-binding factor (CTCF) binding site in HeLa cells (ENCODE: ENCSR000AOA). Importantly, we found this effect to be specific for GBP genes in the cluster in which the cohesin binding site was mutated. *HLA-DRA*, a gene in a different cluster, and *CD74*, a non-clustered memory gene, did not show enhanced priming in this mutant. To confirm this result, we isolated a monoclonal CRISPR line for site C and obtained similar results; loss of site C led to enhanced priming and reinduction specifically of the local *GBP1* and *GBP5* genes (Figure 7C). These results show that, similar to systemic degron-mediated cohesin removal, deletion of a local cohesin binding site leads to an enhanced primed state specifically of GBP genes. Recent work reported that decreased enhancer-promoter proximity is required for robust expression of the sonic hedgehog gene during neuronal differentiation (Benabdallah et al., 2019). We speculate that cohesin acts at the GBP and, possibly, HLA clusters in an analogous manner, restricting local contacts and impeding priming and memory establishment.

In summary, we discovered that IFNγ-mediated priming of cells results in a memory of this event that is maintained through numerous cell division cycles, indicating an active self-propagating process. A key insight from our analysis is that strongly primed genes reside in genomic clusters. Memory is controlled, at least in part, by local cohesin function that restricts IFNγ-induced gene expression output. These findings pave the way to explore further mechanisms and ultimately define the link between interferon priming and memory of innate immune signaling.

## STAR★Methods

### Key Resources Table

**Table:** 

REAGENT or RESOURCE	SOURCE	IDENTIFIER
**Antibodies**		

GBP5	Cell Signaling Technology	Cat#67798; RRID: AB_2799735
GBP5	Abcam	Cat#ab96119; RRID: AB_10678091
IRF1	Cell Signaling Technology	Cat#8478; RRID: AB_10949108
EGFP	Chromotek	Cat#3H9; RRID: AB_10773374
α-TUB	Merck	Cat#T9026; RRID: AB_477593
Anti-rabbit (fluorophore conjugated)	LI-COR	Cat#926-32211; RRID: AB_621843
Anti-mouse (fluorophore conjugated)	Rockland	Cat#610-744-124; RRID: AB_1057600
RNA Pol II	Abcam	Cat#ab817; RRID: AB_306327
HA	Merck	Cat#12CA5; RRID: AB_514505
H3K27ac	Abcam	Cat#ab4729; RRID: AB_2118291
H3K36me3	Abcam	Cat#ab9050; RRID: AB_306966
H3K79me2	Abcam	Cat#ab3594; RRID: AB_303937
H3K4me2	Abcam	Cat#ab32356; RRID: AB_732924
SCC1 (RAD21)	Abcam	Cat#ab992; RRID: AB_2176601

**Bacterial and Virus Strains**		

lentiCRISPR v2	Sanjana et al., 2014; Addgene	Addgene #52961
lentiCRISPR v2-Blast	Addgene	Addgene #83480
psPAX2	Addgene	Addgene #12260
pMD2.G	Addgene	Addgene #12259

**Chemicals, Peptides, and Recombinant Proteins**		

Interferon gamma (IFNγ)	Merck	Cat#SRP3058; CAS: 9008-11-1
Triptolide	Merck	Cat#T3652; CAS: 38748-32-2
Indole-3-acetic acid sodium salt (Auxin, IAA)	Merck	Cat#I5148; CAS: 6505-45-9

**Critical Commercial Assays**		

NEBNext Ultra RNA Library Prep Kit for Illumina	New England Biolabs	Cat#E7530
NEBNext Poly(A) mRNA Magnetic Isolation Module	New England Biolabs	Cat#E7490
NEBNext Ultra II Directional RNA Library Prep Kit	New England Biolabs	Cat#E7760
Nextera XT DNA Library Preparation Kit	Illumina	Cat#FC-131-10
TDE1 Tagment DNA Enzyme	Illumina	Cat#15027865

**Deposited Data**		

Raw and analyzed data, ATAC-seq	this paper	GEO: GSE150199
Raw and analyzed data, ChIP-seq	this paper	GEO: GSE150199
Raw and analyzed data, RNA-seq	this paper	GEO: GSE150199
Raw and analyzed data, RNA-seq_SCC1-AID	this paper	GEO: GSE150199
Raw and analyzed data, single-cell RNA-seq	this paper	GEO: GSE150199
Unprocessed western blots	this paper; Mendeley Data	http://dx.doi.org/10.17632/86yrzx7sfb.2

**Experimental Models: Cell Lines**		

Human: HeLa WT	ATCC	Cat#CCL-2; RRID: CVCL_0030
Human: Hs27 (male primary fibroblasts)	Jonathan Howard lab, Instituto Gulbenkian de Ciência, Portugal	RRID: CVCL_0335
Human: HeLa EGFP::GBP5	this paper	N/A
Human: HeLa H3.3-HA	Bodor et al., 2013	N/A
Human: HeLa Kyoto SCC1-EGFP-AID osTIR1+	Wutz et al., 2017	N/A
Human: HeLa Kyoto SCC1-EGFP-AID osTIR1-	Wutz et al., 2017	N/A
Human: HeLa Kyoto	https://web.expasy.org/cellosaurus/CVCL_1922	RRID: CVCL_1922
Human: HeLa Kyoto site A cohesin mutant polyclonal population 1	this paper	N/A
Human: HeLa Kyoto site A cohesin mutant polyclonal population 2	this paper	N/A
Human: HeLa Kyoto site B cohesin mutant polyclonal population	this paper	N/A
Human: HeLa Kyoto site C cohesin mutant polyclonal population	this paper	N/A
Human: HeLa Kyoto site C cohesin mutant clonal population	this paper	N/A
Human: HCT116	ATCC	CCL-247; RRID: CVCL_0291
Human: hTERT RPE-1	ATCC	CRL-4000; RRID: CVCL_4388
Human: HEK293	ATCC	CRL-1573; RRID: CVCL_0045

**Oligonucleotides**		

RT-qPCR primers (see Table S4)	this paper	N/A
Mutagenesis oligonucleotides (see Table S4)	this paper	N/A

**Recombinant DNA**		

pX330-U6-Chimeric_BB-CBh-hSpCas9	Cong et al., 2013; Addgene	Addgene #42230
EGFP-FLAG-miniAID-FLAG synthetic construct	this paper	N/A

**Software and Algorithms**		

GPP sgRNA Designer software	Doench et al., 2016	https://portals.broadinstitute.org/gpp/public/analysis-tools/sgrna-design
Bwa-mem version 0.7.17.1	Li, 2013	http://bio-bwa.sourceforge.net/
Htseq-count version 0.9.1	Anders et al., 2015	https://htseq.readthedocs.io/en/master/count.html
DESeq2 version 2.11.40.6	Love et al., 2014	http://bioconductor.org/packages/release/bioc/html/DESeq2.html
Bowtie2 version 2.3.4.3	Langmead and Salzberg, 2012	http://bowtie-bio.sourceforge.net/bowtie2/index.shtml
bamCompare	Ramírez et al., 2016	https://deeptools.readthedocs.io/en/develop/content/tools/bamCompare.html

### Resource Availability

#### Lead Contact

Further information and requests for resources and reagents should be directed to and will be fulfilled by the Lead Contact, Lars E.T. Jansen (lars.jansen@bioch.ox.ac.uk).

#### Materials Availability

All unique/stable reagents generated in this study are available from the Lead Contact with a completed Materials Transfer Agreement.

#### Data and Code Availability

The data reported in this paper was deposited in the Gene Expression Omnibus (GEO) database (accession number: GSE150199).

Original data for figures in the paper is available at Mendeley Data http://dx.doi.org/10.17632/86yrzx7sfb.2

### Experimental Model and Subject Details

#### Human Cell Lines

• HeLa (female, RRID: CVCL_0030)
• HeLa EGFP::GBP5
• HeLa H3.3-HA (Bodor et al., 2013)
• Hs27 (male, RRID: CVCL_0335)
• HeLa Kyoto (female, RRID: CVCL_1922)
• HeLa Kyoto SCC1-EGFP-AID osTIR1+ (Wutz et al., 2017)
• HeLa Kyoto SCC1-EGFP-AID osTIR1- (Wutz et al., 2017)
• HeLa Kyoto site A cohesin mutant polyclonal population 1
• HeLa Kyoto site A cohesin mutant polyclonal population 2
• HeLa Kyoto site B cohesin mutant polyclonal population
• HeLa Kyoto site C cohesin mutant polyclonal population
• HeLa Kyoto site C cohesin mutant clonal population
• HCT116 (male, RRID: CVCL_0291)
• hTERT RPE-1 (female, RRID: CVCL_4388)
• HEK-239 (female, RRID: CVCL_0045)

#### Culture Conditions

All cell lines used were grown at 37°C, 5% CO2. HeLa, male primary fibroblasts (Hs27) and HEK293 cells were grown in DMEM containing glucose, glutamine and pyruvate (GIBCO, 41966-029) supplemented with 10% newborn calf serum (GIBCO, 16010-159) and 1% Penicillin-Streptomycin (GIBCO, 15140-122). HCT116 cells were grown in McCoy’s 5a Medium (Modified) containing glutamine (GIBCO, 26600-023) supplemented with 10% Fetal Bovine Serum (GIBCO, 10500-064) and 1% Penicillin-Streptomycin (GIBCO, 15140-122). hTERT RPE-1 cells were grown in DMEM:F12 Medium (GIBCO, 21331-020) supplemented with 10% Fetal Bovine Serum (GIBCO, 10500-064) and 1% Penicillin-Streptomycin (GIBCO, 15140-122). Cells were counted with Scepter Handheld Automated Cell Counter with 60 μm sensors (Merck, PHCC60050). Transfection of cells was performed using Lipofectamine LTX (Thermo Fisher Scientific) according to the manufacturer’s instructions.

### Method Details

#### Reagents

Unless otherwise noted, chemicals used were obtained from Merck and enzymes from New England Biolabs. IFNγ, IFNα and IFNβ were used at a concertation of 50ng/mL. Triptolide was used at concentration of 2nM unless stated otherwise. Auxin: Indole-3-acetic acid sodium salt (IAA) was used at concentration of 500μM. The following antibodies were used in this work: GBP5 (Cell Signaling Technology, 67798; Abcam, ab96119), IRF1 (Cell Signaling Technology, 8478), EGFP (Chromotek, 3H9), α-TUB (Merck, T9026), anti-rabbit (fluorophore conjugated) (LI-COR, 926-32211), anti-mouse (fluorophore conjugated) (Rockland, 610-744-124), RNA Pol II (Abcam, ab817), HA (Merck, 12CA5), H3K27ac (Abcam, ab4729), H3K36me3 (Abcam, ab9050), H3K79me2 (Abcam, ab3594), H3K4me2 (Abcam, ab32356), SCC1 (RAD21) (Abcam, ab992).

#### FACS and Cytometry

For fluorescence-activated cell sorting (FACS) and cytometry, cells were collected by centrifugation for 5 minutes at 500 g, re-suspended in ice-cold Sorting Medium (1% Fetal Bovine Serum in PBS, 0.25mg/mL Fungizone (Thermo Fisher Scientific), 0.25μg∕mL/10μg∕mL Amphotericin B/Gentamicin (GIBCO)) and filtered using 5mL polystyrene round-bottom tubes with cell-strainer caps (Falcon) before sorting (FACSAria) or cytometry analysis (FACSCalibur) (BD Biosciences). For sorting, cells were collected in Conditional Medium (1:1 mixture of fresh complete medium and medium collected from proliferating cell cultures that is 0.45μm filtered, supplemented with 20% Fetal Bovine Serum, 0.25mg/mL Fungizone (Thermo Fisher Scientific), 0.25μg∕mL/10μg∕mL Amphotericin B/Gentamicin (GIBCO)).

#### DNA Constructs and Genome Engineering

To construct the EGFP::GBP5 promoter trap cell line the plasmid pX330-U6-Chimeric_BB-CBh-hSpCas9 (Addgene #42230) (Cong et al., 2013) was employed with two guide RNA (gRNA) sequences: 5′-GCAAAGTAACATCCTAGACA-3′ and 5′-GGCACATGGGGTCTGACATG-3′ targeting exon 2 of the *GBP5* gene that encodes the start codon. The gRNAs were selected using the GPP sgRNA Designer software (Doench et al., 2016). The homology repair template consisted of an EGFP cassette generated from a synthetized DNA EGFP-FLAG-miniAID-FLAG (GeneScript) with 75bp homology arms to the *GBP5* gene added during PCR amplification (Q5 polymerase, NEB). The homology arms span: chr1:89269477-89269552 and chr1:89269555-89269630 of the human hg38 genome. For both guide RNAs the homology arms were designed to introduce a silent mutation in the protospacer-adjacent motif (PAM) recognition sequence after successful repair to prevent Cas9 re-cutting. After transfection of the mixture of the plasmid containing the Cas9/guide RNA and the homology repair template (1:2 ratio) the cell lines treated with different guide RNAs were mixed and grown for 48h. Cells were induced with INFγ and a polyclonal population was sorted based on EGFP fluorescence as described above. After 48h of proliferation without INFγ a polyclonal population was again sorted based on the lack of EGFP signal. This step was performed to remove cells with an improper targeted EGFP cassette. The cells were left to proliferate for 48h, induced again with INFγ and single cell sorted to generate monoclonal lines. Following clonal selection, cells were maintained in culture for about 30 days to erase INFγ priming before being used in experiments.

To construct the GBP gene cluster cohesin site mutants we deleted a ∼1 kbp region using a double Cas9 cut strategy. To achieve this we used a combination of two plasmids: lentiCRISPR v2 (Addgene #52961) (puromycin resistance) and lentiCRISPR v2-Blast (Addgene #83480) (blasticidin resistance) (Sanjana et al., 2014) with gRNAs flanking the selected cohesin sites. The double drug resistance allowed us to select for a dual Cas9 cut on either site of the cohesin site. Two independent guide RNA pairs for the A site (hg38, chr1:88,997,800-88,999,895) were: 5′-AAATTAGTATATCAAAGGGA-3′, 5′- GTCTCATTGCTGTGTTGCCT-3′ and 5′-TAGATCCCTAGAGCAATGTT-3′, 5′- AGCTTCCTTAGAGATTTCCA-3′. Guide RNAs for the B site (hg38, chr1: 89085327-89086509) were: 5′-AAATGTCTATTCAGGATGAG-3′ and 5′-GTTTCCCTCAATAGACCTTG-3′. Guide RNAs for the C site (hg38, chr1:89357816-89359284) were: 5′-ATTCATATCCTGCTCTAGCG-3′ and 5′-ACATTGCTATTGGCATACCT-3′. The gRNAs were selected using the GPP sgRNA Designer software (Doench et al., 2016). The plasmids with cloned gRNAs were co-transfected (as described above) with lentiviral packaging plasmids: psPAX2 (Addgene #12260), pMD2.G (Addgene #12259) into HEK293 cells at a molar ratio of: 4:3:1. 24h later, medium was refreshed and the cells were incubated for 3 days at 37°C. Medium was collected and filtered through a 0.45μm filter to obtain the viral particles. For lentiviral infections HeLa cells were incubated in medium with 8μg/mL of polybrene (Merck) for 1h and infected with a 1:1 mixture of viruses carrying Cas9 with gRNAs flanking a given cohesin site. The cells were left to grow for 48h followed by selection with both blasticidin (1μg/ml) and puromycin (1μg/ml). For the site C mutant a monoclonal line was additionally established by FACS.

#### Transcriptional Memory Assay

Cells were cultured and split into two parallel cultures. One was mock treated with medium and the other with IFNγ for 24h unless stated otherwise. Cells were then washed three times with PBS and trypsinized (GIBCO) to remove residual INFγ and cleave the extracellular domains of plasma membrane proteins, including the INFγ receptor, to stop residual signaling. Fresh medium was added and cells were allowed to proliferate for 48h hours unless stated otherwise. Next IFNγ was added to the primed and naive cells. After 24h cells were washed, trypsinized, centrifuged 500 g for 5 minutes and the pellets were processed for downstream analysis.

#### RT-qPCR

Cell pellets (8^∗^10^6^ cells) were re-suspend in 0.25mL of PBS and 1mL of TRIzol Reagent was added per sample. Cells were lysed by extensive pipetting and incubated for 5 minutes at room temperature. Next, 0.2mL of chloroform was added per sample, mixed and incubated for 3 minutes at room temperature followed by centrifugation at 12000 g for 15 minutes at 4°C. The aqueous phase was mixed with 0.5mL of 100% isopropanol and incubated at room temperature for 10 minutes followed by centrifugation at 12000 g for 10 minutes at 4°C. The supernatant was removed and the pellet was washed with 1mL of 75% ethanol and air-dried for 10 minutes. Next the RNA pellet was re-suspended in 100 μL of RNase-free water and incubated at 60°C for 10 minutes. The residual DNA was removed with DNase I (Roche) and the RNA was purified with RNeasy Mini kit (QIAGEN) according to manufacturer’s instructions. cDNA was prepared using a High-Capacity RNA-to-cDNA Kit (Applied Biosystems) and the libraries were diluted 10 times before qPCR measurements. The qPCR assay was performed with PerfeCTa SYBR Green FastMix ROX (Quanta) using primers (at 300nM concentration): ACTB_F: 5′-CTCTTCCAGCCTTCCTTCCT-3′, ACTB_R: 5′-AGCACTGTGTTGGCGTACAG-3′, GBP5_F: 5′-TTCAATTTGCCCCGTCTGTG-3′, GBP5_R: 5′-AGGCAGTGTTTCAAGTTGGG-3′, HLA-DRA_F: 5′-GAAAGCAGTCATCTTCAGCGTT-3′, HLA-DRA_R: 5′-AGAGGCATTGGCATGGTGATAAT-3′, GBP1_F: 5′-GTGGAACGTGTGAAAGCTGA-3′, GBP1_R: 5′-CAACTGGACCCTGTCGTTCT-3′, CD74_F: 5′-TGGGAGGTGACTGTCAGTTTG-3′, CD74_R: 5′-AGGCTTTTCCATCCTGGTGAC-3′. All experiments were performed in technical and biological triplicates, for every primer pair a calibration curve was determined and a melting curve was measured at the end of the reaction. The qPCR conditions were as follows: 95°C 3 minutes; [95°C 10 s; 59°C 30 s]x50 cycles. From the calibration curve a linear regression was established and the parameters were used to determine the nucleic acids amount according to the equation: N = 10ˆ((Ct - b)/a), N = relative measure of DNA amount; a = slope of regression; b = y intersect point; Ct = qPCR measurement value.

#### Immunoblotting

Cell pellets (8^∗^10^6^ cells) were re-suspended in protein loading buffer (125mM Tris-HCl pH 6.8, 10% Glycerol, 1% SDS, 0.2% (w/v) Orange G, 5% β-mercaptoethanol) and incubated at 95°C for 5 minutes. Benzonase (50U) was added to the lysates and the samples were incubated in room temperature for 30 minutes. Next, sample concentration was normalized based on the 260nm absorbance determined by NanoDrop. Normalized samples were separated on a 12% SDS-PAGE gel (Bio-Rad), transferred to nitrocellulose (Bio-Rad), blocked with Odyssey Blocking Buffer (LI-COR) and incubated overnight with primary antibodies in Odyssey Blocking Buffer. Blots were then washed three times with TBST (20mM Tris-HCl pH 7.5, 150mM NaCl, 0.1% Tween 20) and incubated with secondary antibodies (LI-COR) in Odyssey Blocking Buffer. Blots were next washed three times with TBST and analyzed by Odyssey Imaging System (LI-COR).

#### RNA-seq

For transcriptional memory experiments in HeLa cells, three biological replicates of naive, priming, primed and reinduced cells were processed for RNA isolation as described above for RT-qPCR measurements. Qubit RNA BR Assay Kit (Thermo Fisher Scientific) was used for quantification and RNA ScreenTape (Agilent) for accessing RNA integrity. RNA was then used for polyA positive library preparation with the NEBNext Ultra RNA Library Prep Kit for Illumina (New England Biolabs), according to the manufacturer’s protocol. Sequencing was performed on the NextSeq 500 System with the NextSeq 500/550 v2.0 Kit (Illumina) with 75bp read length in single end mode. Library preparation and sequencing was performed at the EMBL Genomics Core Facility.

For the cohesin depletion experiments, HeLa Kyoto SCC1-AID osTIR1 positive / negative cells (Wutz et al., 2017) were treated or not with auxin for 3h followed by treatment with IFNγ (Merck) or mock medium for 16h. Cells were then washed three times with PBS and trypsinized (GIBCO) to remove residual INFγ and cleave the extracellular domains of plasma membrane proteins, including the INFγ receptor, to stop residual signaling. Part of the culture treated with IFNγ was processed for RNA isolation. Fresh medium was added to the other cultures and cells were allowed to proliferate for 48h. Next IFNγ was added or not to the primed and naive cells. After 24h cells were washed, trypsinized, centrifuged 500 g for 5 minutes and the pellets were processed for downstream analysis. Three biological replicates of naive, priming, primed, reinduced and induced cells were processed for RNA isolation using the TRIzol Reagent. Qubit RNA BR Assay Kit (Thermo Fisher Scientific) was used for quantification and RNA ScreenTape (Agilent) for accessing RNA integrity. Purification of mRNA, generation of double stranded cDNA and library construction were performed using NEBNext Poly(A) mRNA Magnetic Isolation Module and NEBNext Ultra II Directional RNA Library Prep Kit (New England Biolabs) with previously reported barcode tags (Lamble et al., 2013). The concentrations used to generate the multiplex pool were determined by Quant-iT PicoGreen dsDNA Assay Kit (Thermo Fisher Scientific). The material was sequenced on the NovaSeq6000 instrument (Illumina) with 150bp read length in paired end mode. Library preparation and sequencing was performed in the Oxford Genomics Centre, Wellcome Centre for Human Genetics.

For both the experiments mapping was performed with bwa-mem software (Li, 2013) version 0.7.17.1 in Simple Illumina mode to the hg38 human genome assembly. Gene readcounts were estimated with htseq-count software (Anders et al., 2015) version 0.9.1. Parameters: union mode, feature type exon, ID Attribute gene_id. DESeq2 version 2.11.40.6 was used to estimate the differential expression between samples (Love et al., 2014). Note that RNA-seq data is presented as log2 fold change to estimate the expression difference. The log2 fold change values represent the multiplicative factor “x” on a log2 scale, convertible to a linear fold change by 2ˆx. e.g., a log2 fold change of 0.5 equals 2ˆ0.5≈1.4.

#### Single Cell RNA-seq

For the single cell RNA-seq experiment, naive, stimulated or re-stimulated HeLa cells were processed for single cell sorting (one 96 well plate per condition) as described above, with an additional viability staining using SYTOX AADvanced (Thermo Fisher Scientific). Viable single cells were collected to Lysis Buffer (0.8% Triton X-100, 2U/μl RiboLock (Thermo Fisher Scientific)). The material was used for the single cell Smart-Seq2, Nextera XT library preparation according to the original protocol (Picelli et al., 2013) and sequenced on the HiSeq4000 System (Illumina) with 75bp read length in paired end mode. Library preparation and sequencing was performed in the Oxford Genomics Centre, Wellcome Centre for Human Genetics. Mapping was performed with bwa-mem software version 0.7.17.1 in Simple Illumina mode (Li, 2013) to the hg38 human genome assembly. Gene readcounts were estimated with htseq-count software version 0.9.1 (Anders et al., 2015). Parameters: union mode, feature type exon, ID Attribute gene_id. Gene readcounts were corrected with the following procedure: corrected gene readcounts = gene readcounts / (total mapped reads / 1000000).

#### ATAC-seq

The ATAC-seq procedure was adopted from Chen et al. (2016). In brief, 5^∗^10^4^ naive, stimulated or primed cells were collected by centrifugation for 5 minutes at 500 g. Next, the cells were re-suspended in 50μL of ATAC lysis buffer (10mM Tris-HCl pH 7.4; 10mM NaCl; 3mM MgCl_2_; 0.01% Igepal CA-630) and centrifuged immediately for 10 minutes at 500 g at 4°C. The cell pellets were re-suspended in 50μL tagmentation reaction buffer (25μL 2 × TD buffer, 22.5 μl dH2O, and 2.5μL TDE1 (Tn5 enzyme) (Illumina) and incubated in 37°C for 30 minutes. DNA was purified with MinElute PCR Purification Kit (QIAGEN) (10 μL elution).

For preparation of sequencing libraries the purified DNA was amplified using Q5 Hot start DNA polymerase using indexing primers described in Buenrostro et al. (2013). Initial amplification was performed: 98°C 30 s; [98°C 10 s; 63°C 30 s; 72°C 1 minute]x 5 cycles followed by library quantification with qPCR: 95°C 3 minutes; [95°C 10 s; 58°C 30 s; 72°C 1 minute] and additional PCR amplification (conditions as for the initial 5 cycles). The required number of additional cycles was calculated from the qPCR data by determining the number of cycles to reach 50% of the maximum signal. After library amplification and indexing, DNA was purified and size selected with AMPure XP magnetic beads (Beckman Coulter) according to manufacturer’s protocol. After purification the libraries concentration was determined by Qubit dsDNA HS Assay Kit (Thermo Fisher Scientific) according to manufacturer’s protocol. The fragment size was estimated by DNA ScreenTape (Agilent) and the final library quality was measured with KAPA Library quantification kit (Roche) according to manufacturer’s protocol. For sequencing, multiplexed libraries were diluted to 2nM concentration (calculated based the on the KAPA Library quantification kit). The sequencing was performed on a NextSeq 550 System with the NextSeq 500/550 v2.5 Kit (Illumina) with 75bp read length in single end mode. The data was mapped to the human genome version hg38 using bowtie2 software (Langmead and Salzberg, 2012) version 2.3.4.3 in default mode. Coverage bigwig files were generated using bamCompare software (Ramírez et al., 2016) against the input sequencing by subtraction, with 50bp bin size and read count scaling.

#### ChIPmentation

The ChIPmentation procedure was adopted from Schmidl et al. (2015). In brief, 12^∗^10^7^ naive, stimulated or primed cells were collected by centrifugation for 5 minutes at 500 g. Next, the cells were re-suspended in 25 mL of medium supplemented with 1% formaldehyde and incubated for 10 minutes in room temperature. Cross-linking was quenched by addition of 25mL of 125mM glycine in PBS. Cells were harvested by centrifugation, washed two times with 10mL of cold PBS, frozen in liquid nitrogen and kept in −80°C. To fragment chromatin, cell pellets were re-suspended in Lysis Buffer (50mM Tris-HCl pH 7.5; 1% SDS; 10mM EDTA; protease inhibitors (Roche)) and sonicated with Qsonica Q800R2; 10^7^ cells in 0.3mL of buffer per 0.5mL tube with the following conditions: time – 5 minutes, pulse – 30 s ON/OFF, amplitude – 75%. The resulting material was tested for proper sonication by fragment analysis confirming DNA size between 500 and 4000bp. Chromatin samples were aliquoted at 5^∗^10^6^ cells per tube and frozen in −80°C. For a single immunoprecipitation, 25 μL of protein A or G Dynabeads beads (Thermo Fisher Scientific) were washed twice with ChIP Dilution Buffer (20mM Tris-HCl pH 7.5; 0.1% SDS; 1% Triton X-100; 150mM NaCl; 2mM EDTA; protease inhibitors (Roche)). In parallel, the sample was diluted with ChIP Dilution Buffer up to 2mL and clarified at 12000 g for 10 minutes in 4°C. Input DNA was collected from the supernatant (10μL). Samples were incubated with the beads and a given antibody over night at 4°C with rotation. Beads were washed at 4°C as follows: twice with Low Salt Wash Buffer (50mM Tris-HCl pH 7.5; 150mM NaCl; 1mM EDTA; 1% Triton X-100; 0.1% Na-Deoxycholate; protease inhibitors (Roche)); twice with High Salt Wash Buffer (50mM Tris-HCl pH 7.5; 500mM NaCl; 1mM EDTA; 1% Triton X-100; 0.1% Na-Deoxycholate; protease inhibitors (Roche)); twice with LiCl Wash Buffer (10mM Tris-HCl pH 7.5; 0.5% NP-40; 0.5% Na-Deoxycholate; 250mM LiCl; 1mM EDTA; protease inhibitors (Roche)); twice with TE Buffer (10mM Tris-HCl pH 7.5; 1mM EDTA; protease inhibitors (Roche)) and finally twice with 10mM Tris-HCl pH 7.5. Beads were re-suspended in 30 μL of ChIPmentation reaction buffer (10 mM Tris-HCl pH 8.0; 5mM MgCl_2_; 1 μL TDE1 (Tn5 enzyme) (Illumina). In parallel 1 μL of input was diluted in 29 μL of ChIPmentation reaction buffer. The samples were incubated in 37°C for 10 minutes followed by washing twice in ice-cold LiCl Buffer and TE Buffer. Next, the beads were suspended in 200μl of Elution Buffer (50mM Tris-HCl pH 7.5; 1% SDS; 10mM EDTA; protease inhibitors (Roche)). In parallel the input reaction was mixed with 170 μL of the Elution Buffer. All samples were incubated at 65°C overnight for decrosslinking followed by treatment with PureLink RNaseA (Thermo Fisher Scientific) and Proteinase K. The DNA was purified with MinElute PCR Purification Kit (QIAGEN) (10 μL elution). Library preparation and sequencing was performed as described above for ATAC-seq.

### Quantification and Statistical Analysis

RT-qPCR and RNA-seq data were collected in triplicate. Standard deviation is reported.

Statistical analyses and P value calculations for the RT-qPCR data were performed by two-tailed unpaired t tests (GraphPad Prism 6.01) and can be found in the figure legends.

RNA-seq data statistics were calculated by the DESeq2 software (Love et al., 2014): pvalues are calculated by the Wald test, padj values are calculated by the Benjamini-Hochberg method.

## Supporting Citations

The following references appear in the Supplemental Information: Wang et al., 2018.

## Supplemental Information

Document S1. Figures S1–S7

Table S1. List of All Genes with the Indicated Degree of Priming; i.e., Log2 Fold Change of IFNγ-Induced (Priming) Relative to Naive Cells, Related to Figure 1B

Table S2. List of All Genes with the Indicated Degree of Transcriptional Memory; i.e., Log2 Fold Change of IFNγ Priming Relative to Reinduced Cells, Related to Figure 1C

Table S3. List of All Genes with the Indicated Persistence of mRNA Levels 48 h after Removal of IFNγ; i.e., Log2 Fold Change of Primed Relative to Naive Cells, Related to Figure 4C

Table S4. DNA Oligonucleotides Used in This Work, Related to STAR Methods

Document S2. Article plus Supplemental Information
