# Supplementary material for: Activation of Clustered IFNγ Target Genes Drives Cohesin-Controlled Transcriptional Memory
Source: Mol Cell. 2020 Nov 5;80(3):396–409.e6. doi: 10.1016/j.molcel.2020.10.005 (PMC7657446; doi:10.1016/j.molcel.2020.10.005)
Supplement: Document S1. Figures S1–S7 [file mmc1.pdf]

Molecular Cell, Volume *80*

Supplemental Information

Activation of Clustered IFNγ Target Genes Drives Cohesin-Controlled Transcriptional Memory

Wojciech Siwek, Sahar S.H. Tehrani, João F. Mata, and Lars E.T. Jansen

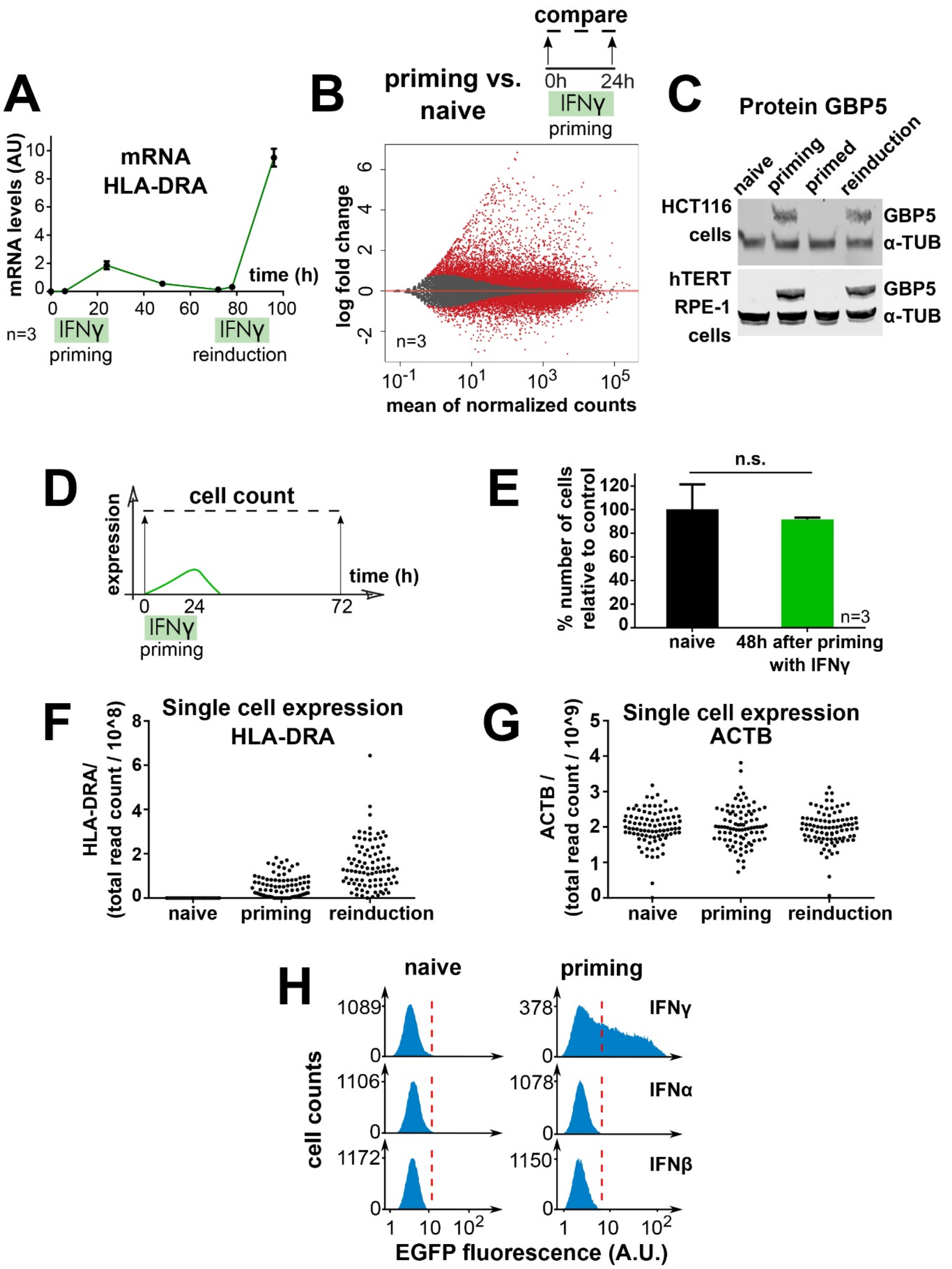

Supplementary Figure 1. Transcriptional memory is cell type dependent and is not a consequence of cell selection. Related to Figure 1. (A) HeLa cells were primed and reinduced according to the regime outlined in Figure 1B. *HLA-DRA* mRNA levels were quantified by RT-qPCR and normalized to *ACTB* expression. Error bars SD, n=3 biological replicates. **(B)** Average read counts for three replicate RNA-seq experiments as outlined in Figure 1B were

assembled for each gene for naïve cells and after priming. The log2 fold change of gene read count following priming over those from naïve cells is plotted. “0” indicates no change while positive or negative values indicate responsiveness to IFNγ. Data was ranked according to the mean expression level for both of the conditions and all replicates. Red dots represent genes with an adjusted P value below 0.1. **(C)** HCT116 and hTERT RPE-1 cells were subjected to IFNγ treatment regime as outlined in Figure 1B, processed for western blotting and probed for GBP5 protein expression. α-tubulin (α-TUB) loading control. **(D)** Experimental outline to assay the effect of IFNγ exposure on HeLa cell proliferation. **(E)** Results from experiment shown in panel D. Cells were primed with IFNγ for 24h and left to proliferate for 48h analogous to experiments in Figure 1B, followed by counting of live cells. The results were normalized to naïve cells. Error bars SD, n=3 biological replicates. **(F, G)** Single cell RNA-seq data from the experiment shown in Figure 3B for *HLA-DRA* (F) and *ACTB* (G) in naïve (N=90), priming (N=89) and reinduction (N=91) states. Every dot represents one cell. **(H)** Experiment to determine cytokine-specific induction of the EGFP::GBP5 promoter trap knock-in cells. Cytometry measurements of EGFP expression before and after stimulation with IFNγ, α and β. Cell frequencies as a function of EGFP fluorescence intensity are plotted.

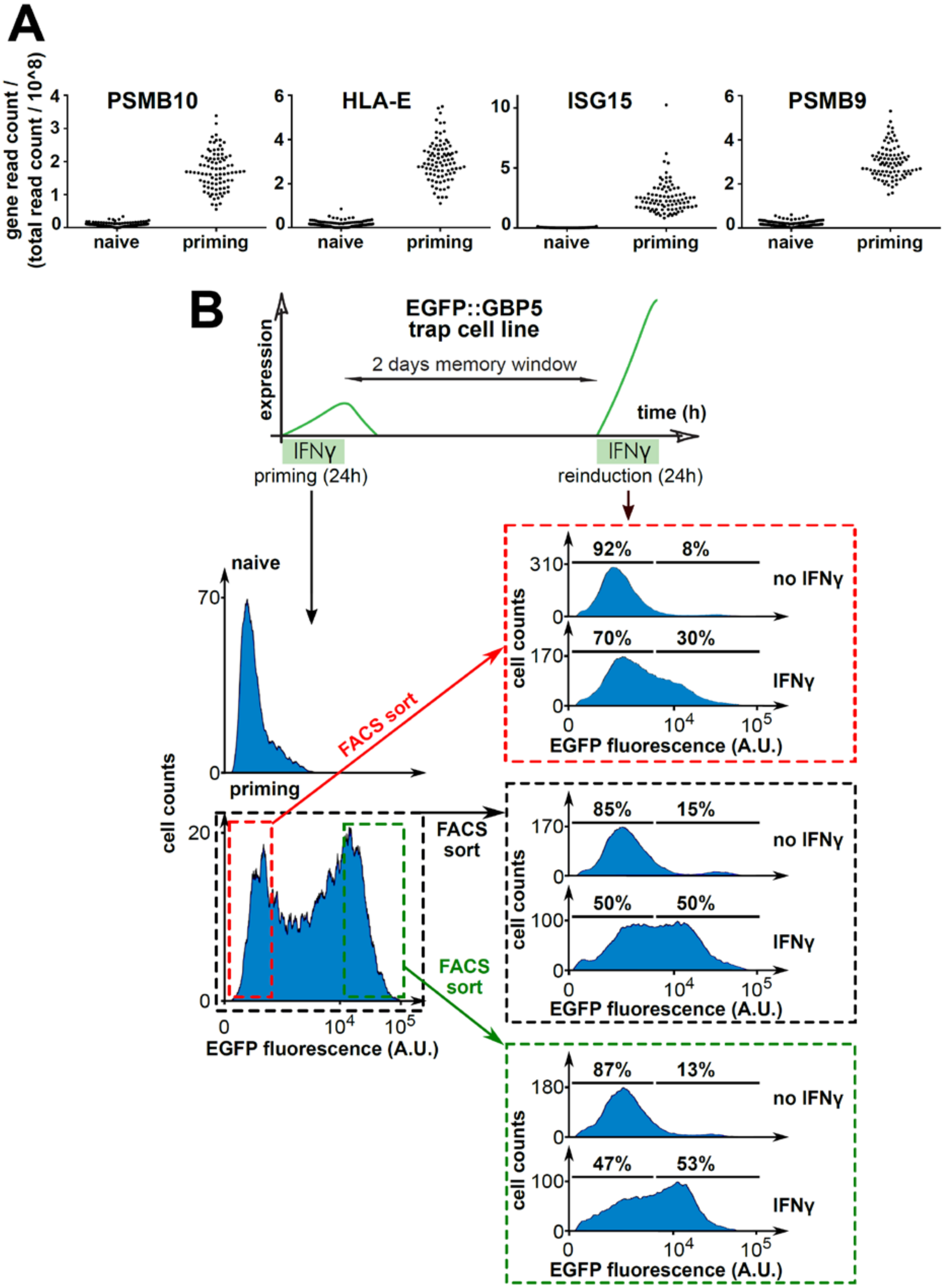

Supplementary Figure 2. HeLa cell population is uniformly responsive to IFNγ and transcriptional output correlates with establishment of transcriptional memory for the *GBP5* gene. Related to Figure 3. (A) Representation of the single cell RNA-seq data in HeLa

cells as in Figure 3C for naïve and IFNγ exposed cells for indicated IFNγ inducible genes. Every dot represents the expression level for the indicated gene for one cell in naïve (N=90) and priming states (N=89). **(B)** HeLa EGFP::GBP5 cells were subjected to IFNγ treatment regime as outlined in the top panel. After priming, a sample from 20% of top expressing, 20% of bottom expressing and all cells were isolated by fluorescence-activated cell sorting (FACS) and left to proliferate for 48h. Cells were then reinduced or not with IFNγ and subjected to cytometry to assay EGFP expression. Cell frequencies as a function of EGFP fluorescence intensity are plotted. Cut-off for cell percentages expressing or not expressing are based on the "all cell" sort IFNγ stimulation.

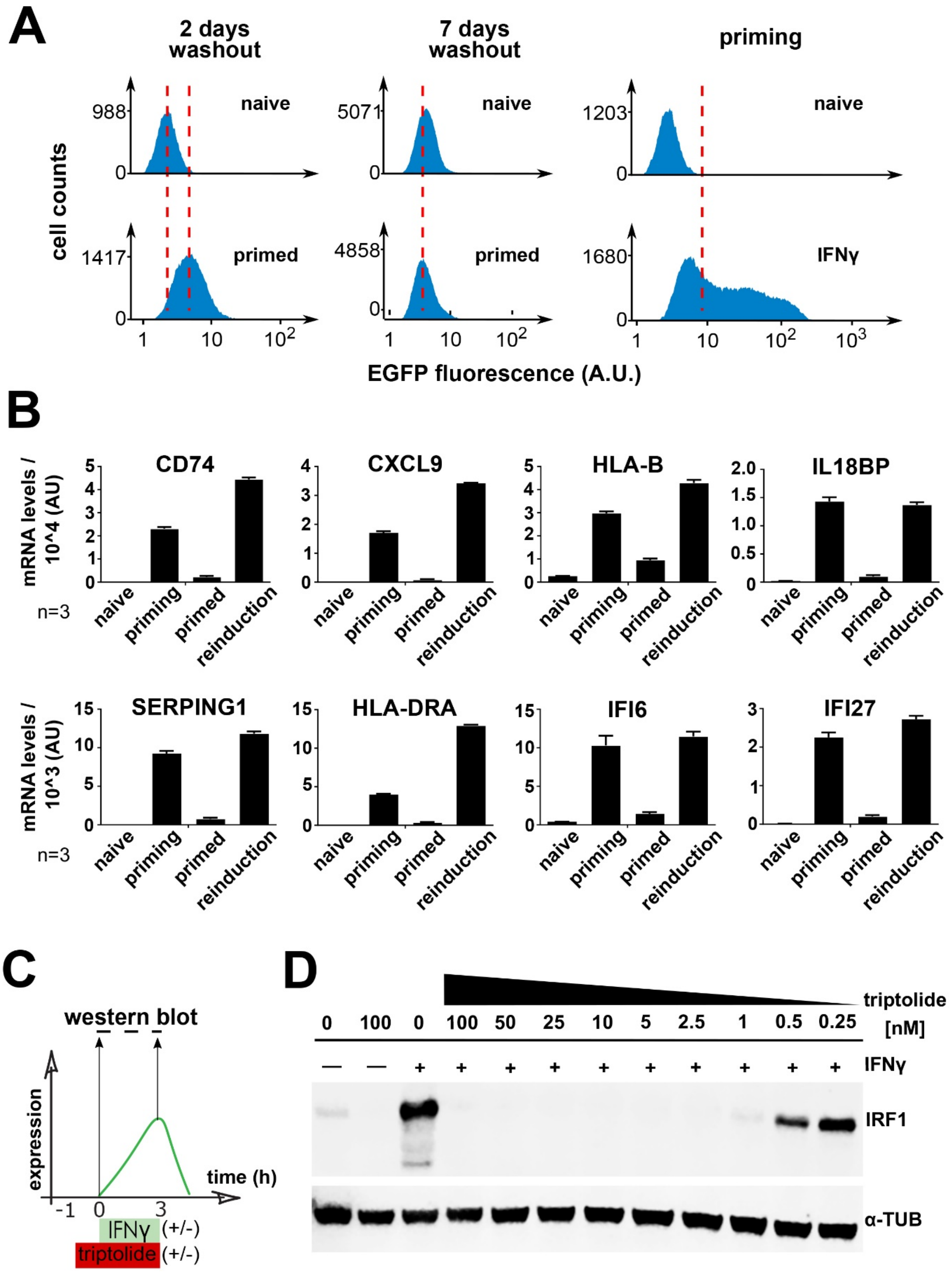

Supplementary Figure 3. Absence of ongoing transcription during maintenance of transcriptional memory. Related to Figure 4. (A) EGFP::GBP5 reporter cells were subjected to IFNγ treatment regime as outlined in Figure 4A and processed for cytometry. Cell frequencies as a function of EGFP fluorescence intensity are plotted. Red dotted lines are fiducial marks to aid comparison. **(B)** HeLa cells were subjected to IFNγ treatment regime and

RNA-seq as outlined in Figure 1B, C. Expression levels of genes that show elevated mRNA in primed over naïve cells (see Figure 4C) were plotted (Error bars SD, n=3 biological replicates). **(C)** Outline of experiment to determine the minimal inhibitory concentration of triptolide impacting expression of IFNγ responsive genes. **(D)** HeLa cells were subjected to IFNγ and triptolide titration series as outlined in panel C, processed for western blotting and probed for an IFNγ early response gene – IRF1 expression. α-tubulin (α-TUB) loading control.

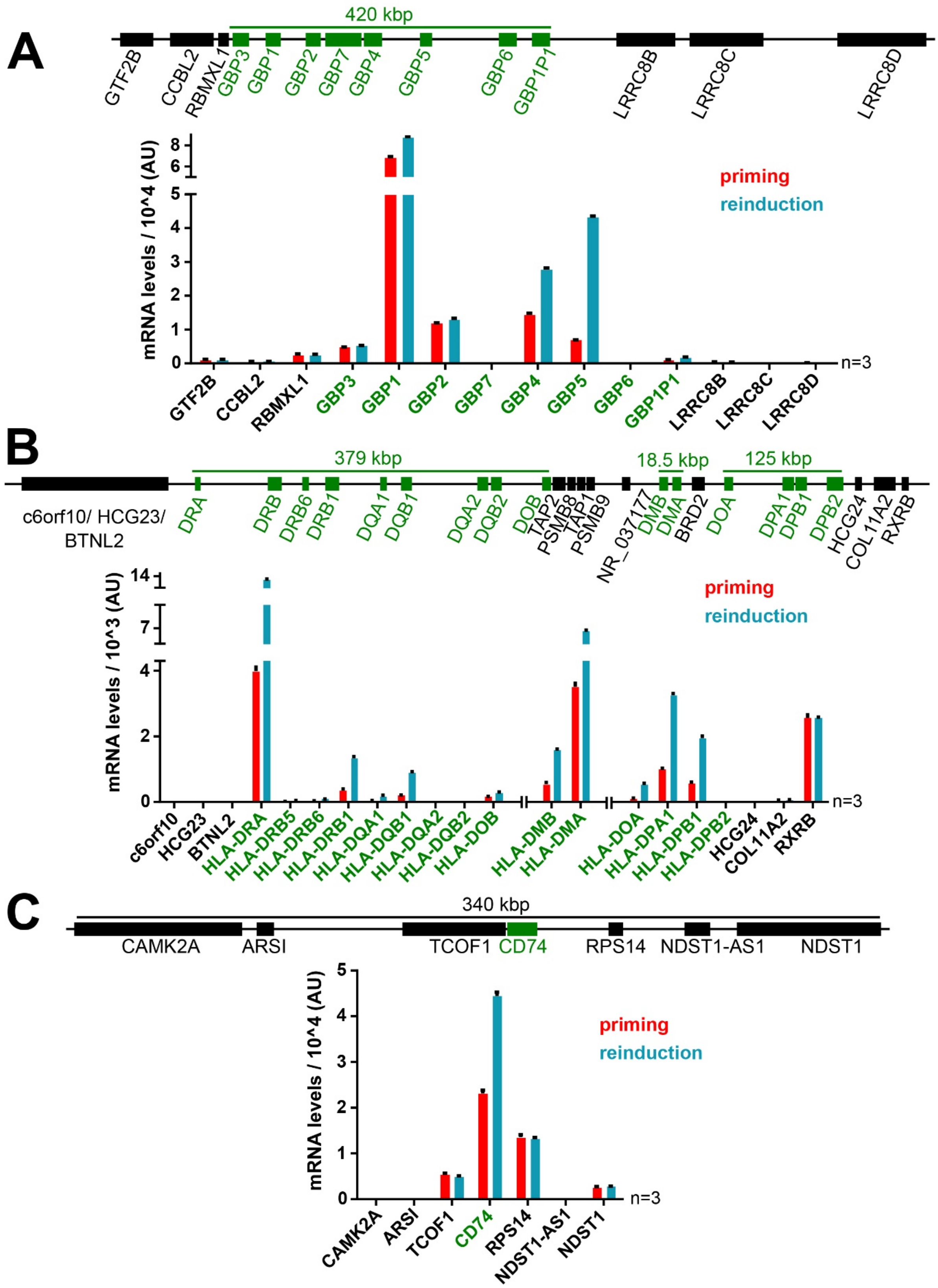

Supplementary Figure 4. The two major classes of genes that are primed for re-expression, GBPs and HLAs, reside in genomic gene clusters. Related to Figure 6. (A) Representation of the genomic structure of the GBP locus and the processed RNA-seq data from experiment shown in Figure 1B. Read counts were assembled for each gene of the GBP

cluster (green) and three flanking genes (black) following priming and reinduction. **(B)** As in A, but for the HLA gene cluster. **(C)** As in A, but for the non-cluster *CD74* gene. Error bars SD, n=3 biological replicates.

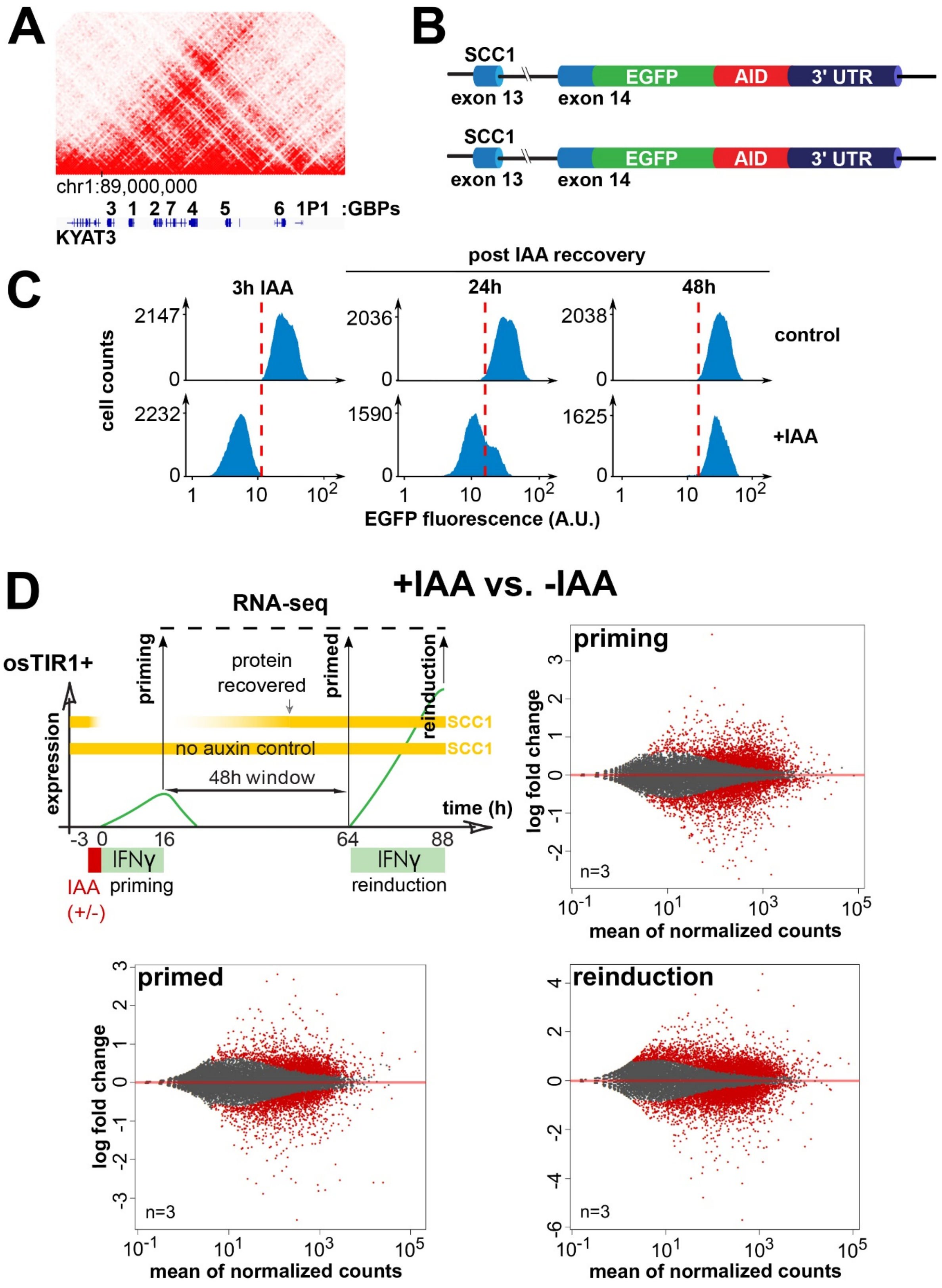

Supplementary Figure 5. Auxin-mediated depletion of the cohesin subunit SCC1 is complete in 3 hours, fully recovers within 48 hours and has broad effects on gene expression. Related to Figure 6. (A) Published Hi-C dataset showing *GBP* genes clustered in a single topologically associating domain (TAD) in the GM12878 cell line with 5kbp resolution

(Rao et al., 2014), the data was visualized with the 3D Genome Browser (Wang et al., 2018). **(B)** Schematic of *SCC1* genomic structure in HeLa Kyoto SCC1-EGFP-AID cell line. Exon 14 bearing the end of the *SCC1* ORF is tagged in frame with *EGFP* and an AID-degron. Both alleles are modified (Wutz et al., 2017). **(C)** Cytometry analysis of EGFP expression in the SCC1-EGFP-AID cells after 3h depletion with auxin (IAA) and following 24h and 48h washout of auxin. Cell frequencies as a function of EGFP fluorescence intensity are plotted. **(D)** Representation of processed RNA-seq data from HeLa Kyoto, SCC1-AID, osTIR1 positive cells as described in upper left panel (identical to Figure 6A). Average read counts for three replicate experiments were assembled for each gene following control (-IAA) or auxin treatment (+IAA). The log2 fold change of gene read counts after IAA treatment over mock treated cells is plotted following priming, in primed cells and after reinduction. “0” indicates no change while positive or negative values indicate increased or decreased expression upon IAA treatment, respectively. Data was ranked according to the mean expression level for both of the conditions and all replicates. Red dots represent genes with an adjusted P value below 0.1.

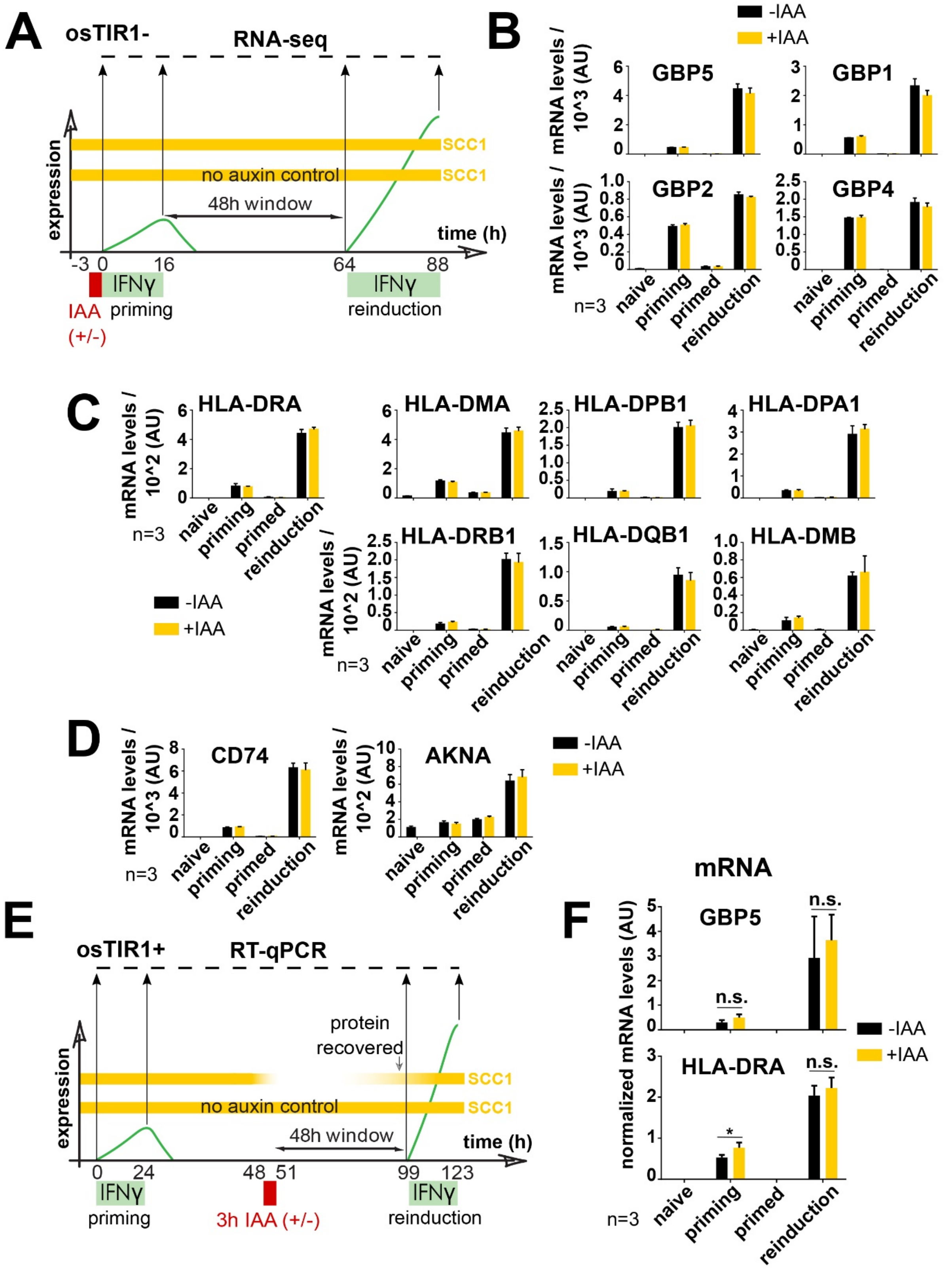

Supplementary Figure 6. Effects on establishment of transcriptional memory following auxin addition to HeLa SCC1-AID cells are dependent on cohesin depletion; and cohesin is not required for maintenance of transcriptional memory of the *GBP5* and *HLA-DRA* genes. Related to Figure 6. (A) Outline of a transcriptional memory experiment (analogous to

Figure 6A) but in cells lacking the osTIR1 E3 ligase, analyzed by RNA-seq. **(B)** HeLa Kyoto SCC1-EGFP-AID osTIR1 negative cells were subjected to IFNγ and auxin treatment regime as outlined in panel A. mRNA levels of GBP cluster genes were quantified by RNA-seq in auxin or mock treated cells. Error bars SD, n=3 biological replicates. **(C)** Data presented as in B, but for the HLA cluster genes. **(D)** Data presented as in B, but for the *CD74* and *AKNA* non-cluster genes. **(E)** Outline of a transcriptional memory experiment (analogous to Figure 6A) combined with auxin-mediated transient depletion of SCC1-EGFP-AID in HeLa Kyoto osTIR1 positive cells. Cells were treated with auxin to remove SCC1 protein, or left untreated after priming but before reinduction (during the memory window). **(F)** Cells were subjected to IFNγ and auxin treatment regime as outlined in E and processed for RT-qPCR of *GBP5* and *HLA-DRA*, normalized to *ACTB* expression. Error bars SD, n=3 biological replicates.

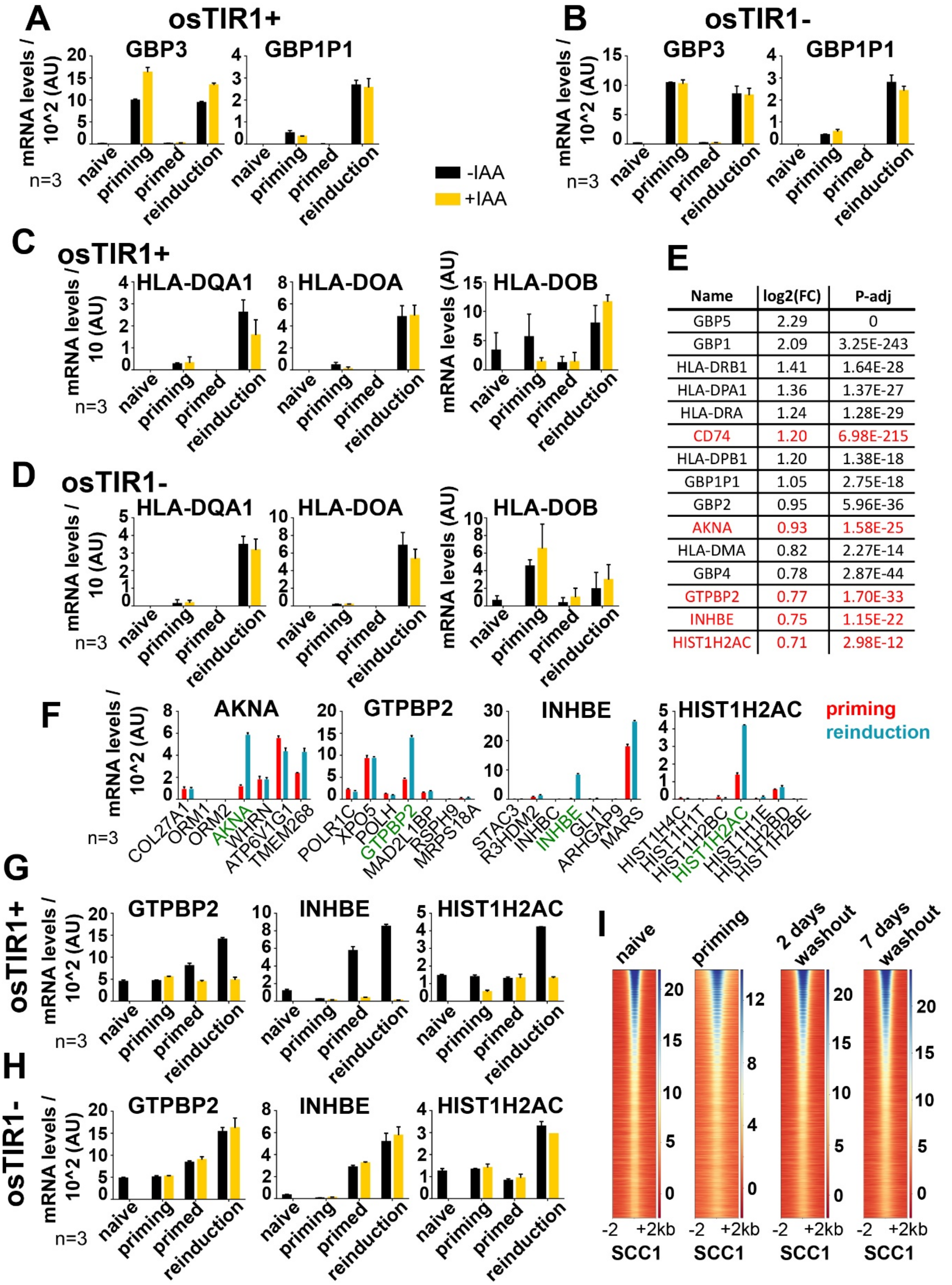

Name	log2(FC)	P-adj
GBP5	2.29	0
GBP1	2.09	3.25E-243
HLA-DRB1	1.41	1.64E-28
HLA-DPA1	1.36	1.37E-27
HLA-DRA	1.24	1.28E-29
CD74	1.20	6.98E-215
HLA-DPB1	1.20	1.38E-18
GBP1P1	1.05	2.75E-18
GBP2	0.95	5.96E-36
AKNA	0.93	1.58E-25
HLA-DMA	0.82	2.27E-14
GBP4	0.78	2.87E-44
GTPBP2	0.77	1.70E-33
INHBE	0.75	1.15E-22
HIST1H2AC	0.71	2.98E-12

Supplementary Figure 7. Non-cluster genes, *GBP* genes at cluster borders and some *HLA* cluster genes lack enhanced memory establishment after cohesin depletion. Related to Figure 6 and 7. (A) HeLa Kyoto, SCC1-AID, osTIR1 positive cells were subjected to IFNγ treatment regime as outlined in Figure 6A. mRNA levels of indicated *GBP* genes were

quantified by RNA-seq. Error bars SD, n=3 biological replicates. **(B)** As in A, but for HeLa Kyoto, SCC1-AID osTIR1 negative cells. **(C, D)** as in A and B, but for indicated *HLA* cluster genes. **(E)** Top memory genes as identified in the HeLa Kyoto, SCC1-AID, TIR1 positive cells by DESeq2 software based on experiment in Figure 6A. Genes not associated in the GBP or HLA clusters are indicated in red. Log2(FC) – log2 fold change between priming and reinduction. P-adj – adjusted P value. **(F)** Representation of processed RNA-seq data from experiment shown in Figure 6A for top non-GBP or -HLA cluster memory genes. Read counts were assembled for indicated genes (labeled in green) and three flanking genes (labelled in black) following priming and reinduction. The experiment was performed in biological triplicate in HeLa SCC1-EGFP-AID osTIR1 positive cells without IAA addition. Error bars SD. **(G)** HeLa Kyoto, SCC1-AID, osTIR1 positive cells were subjected to IFNγ and IAA treatment regime as outlined in Figure 6A. Plotted mRNA levels were quantified by RNA-seq (Error bars SD, n=3 biological replicates). **(H)** As in G but for HeLa Kyoto, SCC1-AID osTIR1 negative cells. **(I)** Heat map analysis of SCC1 genome-wide occupancy in naïve cells, after priming and at 2 and 7 days after IFNγ washout. SCC1 binding sites were ranked based on SCC1 signal in naïve cells. False color scale represents SCC1 occupancy corrected for input.
